# Insights into the genetic foundation of aggression in *Papio* and the evolution of two length-polymorphisms in the promoter regions of serotonin-related genes (*5-HTTLPR* and *MAOALPR*) in Papionini

**DOI:** 10.1186/s12862-016-0693-1

**Published:** 2016-06-10

**Authors:** Urs Kalbitzer, Christian Roos, Gisela H. Kopp, Thomas M. Butynski, Sascha Knauf, Dietmar Zinner, Julia Fischer

**Affiliations:** Cognitive Ethology Laboratory, German Primate Center (DPZ), Leibniz Institute for Primate Research, Kellnerweg 4, 37077 Göttingen, Germany; Department of Anthropology and Archaeology, University of Calgary, 2500 University Dr NW, Calgary, AB T2N 1N4 Canada; Gene Bank of Primates and Primate Genetics Laboratory, German Primate Center (DPZ), Leibniz Institute for Primate Research, Kellnerweg 4, 37077 Göttingen, Germany; Department of Biology, University of Konstanz, 78457 Constance, Germany; Department of Migration and Immuno-Ecology, Max Planck Institute for Ornithology, Am Obstberg 1, 78315 Radolfzell, Germany; Lolldaiga Hills Research Programme, Sustainability Centre Eastern Africa, P. O. Box 149, Nanyuki, 10400 Kenya; Work Group Neglected Tropical Diseases, Pathology Unit, German Primate Center (DPZ), Leibniz Institute for Primate Research, Kellnerweg 4, 37077 Göttingen, Germany

**Keywords:** 5-HT, Behavioral genetics, Candidate gene, Primate, *MAOA-uVNTR*, Neurotransmitter, Baboon, Macaque, *Macaca*

## Abstract

**Background:**

Aggressive behaviors are an integral part of competitive interactions. There is considerable variation in aggressiveness among individuals both within and among species. Aggressiveness is a quantitative trait that is highly heritable. In modern humans and macaques (*Macaca* spp.), variation in aggressiveness among individuals is associated with polymorphisms in the serotonergic (5-HT) neurotransmitter system. To further investigate the genetics underlying interspecific variation in aggressiveness, 123 wild individuals from five baboon species (*Papio papio*, *P. hamadryas*, *P. anubis*, *P. cynocephalus*, and *P. ursinus*) were screened for two polymorphisms in promoter regions of genes relevant for the 5-HT system (*5-HTTLPR* and *MAOALPR*).

**Results:**

Surprisingly, despite considerable interspecific variation in aggressiveness, baboons are monomorphic in *5-HTTLPR*, except for *P. hamadryas*, which carries one additional allele. Accordingly, this locus cannot be linked to behavioral variation among species. A comparison among 19 papionin species, including nine species of macaques, shows that the most common baboon allele is similar to the one described for Barbary macaques (*Macaca sylvanus*), probably representing the ancestral allele in this tribe. It should be noted that (almost) all baboons live in Africa, but within *Macaca* only *M. sylvanus* lives on this continent. Baboons are, however, highly polymorphic in the so-called ’warrior gene’ *MAOALPR*, carrying three alleles. Due to considerable variation in allele frequencies among populations of the same species, this genotype cannot be invoked to explain variation in aggressiveness at the species level.

**Conclusions:**

This study provides another indication that *5-HTTLPR* is not related to aggressiveness in primates *per se*, but may have been under differential selective pressures among taxa and potentially among populations in different geographic regions. The results on *MAOALPR* alleles in *Papio* indicate that variation in the metabolism of monoamine neurotransmitters and associated behaviors is more important among populations than among species. We, therefore, propose to compile behavioral data from additional populations of *Papio* to obtain further insight into the genetics underlying behavioral differences among primate species.

**Electronic supplementary material:**

The online version of this article (doi:10.1186/s12862-016-0693-1) contains supplementary material, which is available to authorized users.

## Background

### Genetics of aggression

In order to maximize their fitness, individuals compete for resources, including mates. In this context, aggressive behaviors, which are commonly defined as physical attacks and the threat of such attacks [1] play a key role. Escalated aggression, however, may incur high costs such as fatal injury. According to Anholt and Mackay [2], intermediate levels of aggressiveness should, therefore, be favored. As such, aggressiveness is assumed to be under stabilizing selection [2]. Nevertheless, variation in aggression can be observed on different levels. In addition to short-term variation within individuals (*e.g.* in relation to context), there are relatively stable long-term differences among individuals (*i.e.* personality differences, *e.g*. [3]) and among closely related species (*e.g.* [4–6]).

Within many species, including modern humans (*Homo sapiens*) [3] and non-human primates [7], heritability estimates for inter-individual differences in aggressiveness are generally high [2]. The strong genetic component has been demonstrated by experiments in which highly aggressive or docile individuals have been bred within a few generations, for example in house mouse (*Mus musculus*) [8] or in silver fox (*Vulpes vulpes*) [9]. Aggressiveness constitutes a quantitative trait affected by multiple genes, but the specific combination of genes involved in aggressiveness is not clear [2].

Several hormone and neurotransmitter systems are assumed to affect aggressiveness. If so, corresponding genes may be involved in the regulation of associated behaviors [10, 11]. One neurotransmitter system associated with aggressiveness and impulsiveness in mammals is the brain serotonin (5-HT) system (reviewed in [12, 13]). Research on rhesus macaques (*Macaca mulatta*) indicates that individual differences in 5-HT activity are heritable [14, 15] and stable over time [16–18]. This makes the 5-HT system a good candidate to be associated with genetic-based stable differences in aggressiveness among individuals or species.

The 5-HT transporter (5-HTT) and monoamine oxidase A (MAOA) are two important proteins regulating the 5-HT system. 5-HTT is responsible for the re-uptake of 5-HT from the synaptic cleft, while MAOA oxidizes 5-HT to its metabolite 5-hydroxyindoleacetic acid (5-HIAA). Accordingly, variants of genes encoding for these proteins may affect variation in aggressiveness. Regulatory regions (*i.e*. promoters) are of particular interest as they determine the transcription profile of a gene [19]. While mutations in coding regions can affect the functionality of a gene product, including inactivation, mutations in promoter regions may only affect transcriptional activity. Hence, promoter regions appear to be especially suitable targets for natural selection acting on quantitative traits [19], such as aggressiveness.

For the *5-HTT* gene (*SLC6A4*), a functional length-polymorphism in the promoter region, the *5-HTT*-linked polymorphic region (*5-HTTLPR*) is well documented in humans [20], as well as in several species of apes and Old World monkeys [21–23]. Variation in length is caused by a variable number of 21–23 base pairs (bp)-repeat elements. Hominoids (humans and apes) vary at polymorphic locus 1 (PL1), though there is some variation among hominoid species for the specific position of this locus [23]. Macaques (*Macaca* spp.), in contrast, vary at polymorphic locus 2 (PL2) [21]. The *5-HTTLPR* genotype appears to affect the in-vitro transcription rate (humans [20]; *M. mulatta* [24]), and various behaviors (humans *e.g.* [25, 26]; *M. mulatta e.g.* [27, 28]). As aggression is thought to be linked to 5-HT activity, the effect of the *5-HTTLPR* genotype on aggressiveness has been investigated (*e.g.* [28]). The results, however, are inconsistent, and recent meta-analyses did not resolve the involvement of this polymorphism in aggressiveness [29, 30].

In the same context, the effects of variants of the *MAOA* gene have been intensively studied. Brunner et al. [31] reported on a Dutch family carrying a nonsense mutation in the *MAOA* gene that resulted in an extremely aggressive phenotype in males [31]. Importantly, the gene is located on the X-chromosome; males only possess one copy whose disruption leads to a complete inactivation of MAOA. The behavioral consequences of this disruption were confirmed by ‘knock-out’ experiments in mice which resulted in a similar increase in male aggressiveness [32–34]. These observations indicate the importance of the *MAOA* gene for the regulation of aggression, but genetic variants must be more common than such rare (and disruptive) non-sense mutations in order to be linked to common variation in behavior.

The *MAOALPR* (or *MAOA-uVNTR*) represents such a common and important polymorphism in primates [35–37]. Similar to *5-HTTLPR,* this polymorphism consists of a variable number of repeats within the promoter region of the *MAOA* gene. The consensus sequence varies among species (18–30 bp), and the number of repeats differs both among and within many species [37–39]. In humans [35] and in *M. mulatta* [36], different alleles result in different in-vitro transcription rates and appear to have an impact on aggressiveness and impulsiveness (humans [40]; *M. mulatta* [36, 41]; review [42]; meta-analyses [29, 30]). These observations gave rise to the nickname ‘warrior gene’ for the *MAOA* gene (*e.g.* [43]).

### Interspecific behavioral variation in macaques in relation to 5-HTTLPR and MAOALPR genotypes

At the individual level, the effects of *5-HTTLPR* and *MAOALPR* genotypes on aggressiveness do not appear to be simple additive genetic effects. Instead, both *5-HTTLPR* [28] and *MAOALPR* [36, 40] affect variation in aggressiveness depending on early experiences [*i.e.* genetic x environment interaction (G × E)]. At the species level, in contrast, variation in aggressiveness may be more generally linked to different genotypes at these loci. This has been suggested for different species of macaques, which vary in their degree of tolerance [6], possibly related to the distribution of different alleles in *5-HTTLPR* and *MAOALPR* [38, 44, 45]. It should be noted that (1) most samples used in these studies came from captive individuals [38], whose genetic composition may not reflect a natural population, and (2) that sample sizes ranged from two to several hundred individuals per species.

### Interspecific behavioral variation among baboons

Baboons (*Papio* spp.) are well suited to further investigate the genetic differences underlying variation in aggressiveness because the species, although closely related, show considerable variation in social behavior. Furthermore, they are closely related to macaques (both belong to the tribe Papionini) and, therefore, likely to show similar polymorphisms.

**Fig. 1:**
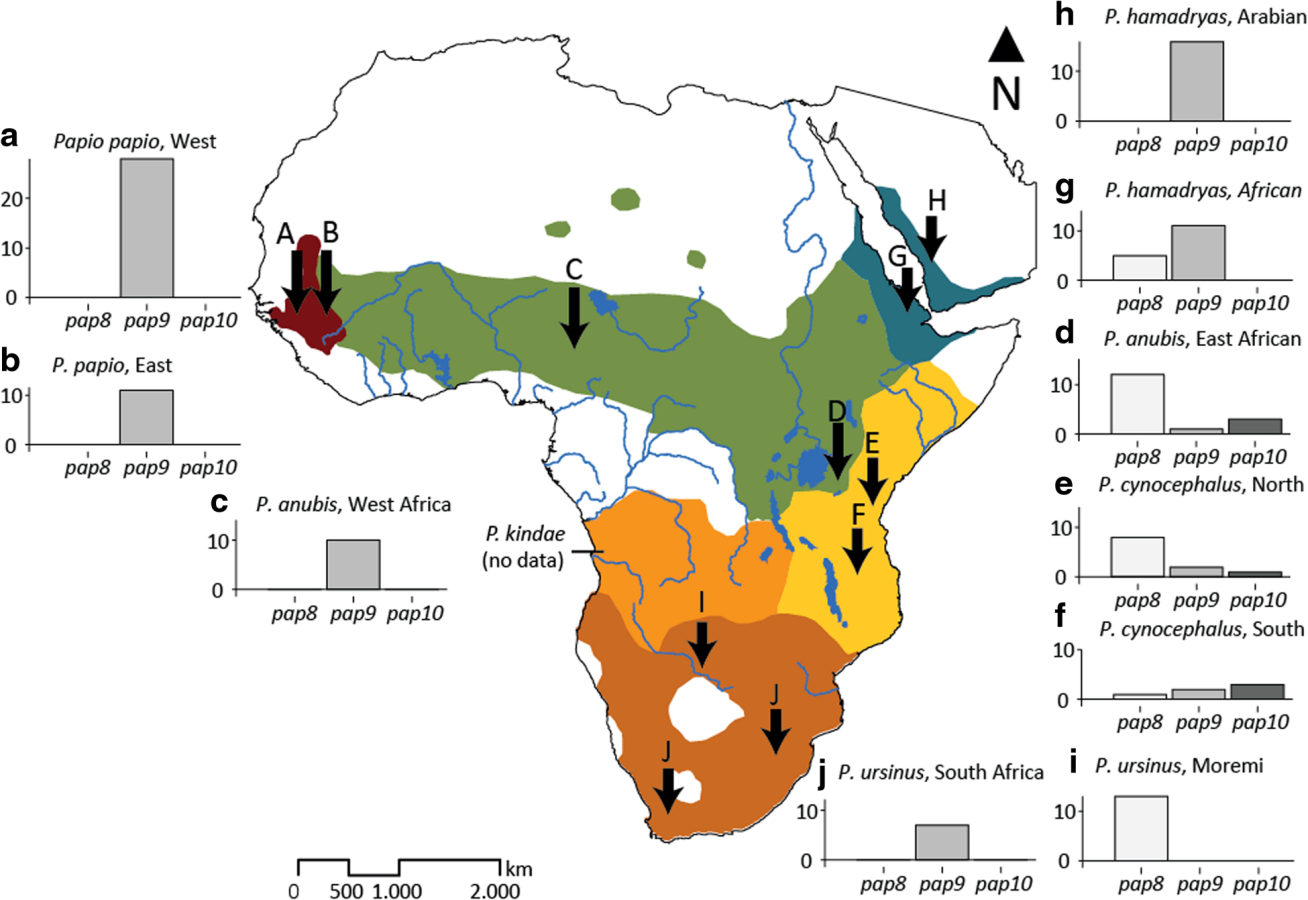
Geographical distribution of baboon species (*Papio* spp.) and *MAOALPR* allele frequencies of different populations. The bar plots show the frequency of *pap8*, *pap9*, and *pap10*-alleles for all sampled populations, labeled with letters **a**-**j** (for sample locations see Additional file 1: Table S1). Populations of the same species can vary considerably. Generally, frequencies of *pap8* decrease and *pap9* increase from populations in the south to populations in the north-west and north-east. South Africa *P. ursinus* does not adhere to this pattern, and southern *P. cynocephalus* mainly carries *pap10*. The geographical distribution of *Papio* spp. is adapted from Zinner et al. [46]. Kinda baboons (*P. kindae*), which are often recognized as a subspecies of *P. cynocephalus*, were not included in this study

*Papio* is thought to have originated in southern Africa, and started to disperse across Africa around 2 million years ago (mya) [46]. At present, baboons inhabit large parts of sub-Saharan Africa and a small part of Arabia (Fig. 1). Commonly, *Papio* is divided into six morphotypes [46], which should be classified as species according to the phylogenetic species concept – although they commonly hybridize in contact zones (*e.g.* [47–49]). In accordance with recent literature [50] we recognize the morphotypes as species.

Behavioral observations suggest that baboons show a gradient of decreasing male aggressiveness and increasing male tolerance from southern to northern species [5, 51, 52]. In southern African chacma baboons (*P. ursinus*) and in the more northern yellow baboons (*P. cynocephalus*) and olive baboons (*P. anubis*), males show intense contest competition over mates [53–55], and fights often lead to serious injuries [56–58]. Yellow and olive baboon males appear to show a higher spatial tolerance, potentially linked to the occurrence of coalitions in these two species (*e.g.* [59, 60]). In hamadryas baboons (*P. hamadryas*) of north-eastern Africa, fights among males are highly ritualized and rarely result in injuries [61]. Males not associated with females show a high spatial tolerance but once they associate with females they become less tolerant [61, 62]. Finally, male Guinea baboons (*P. papio*) of north-western Africa, show high tolerance, as well as low frequency and intensity of fights [5, 63–65].

Females of different baboon species also vary with regard to their social behavior. Females of *P. ursinus*, *P. cynocephalus* and *P. anubis* show highly despotic social relationships [66–68], while female behavior in *P. hamadryas* and *P. papio* is mainly male-directed and females rarely interact with each other (*P. hamadryas* [61], but see [69]; *P. papio* [70]; UK pers. obs.), which includes the absence of frequent aggressive interactions among females. Thus, variation in female behavior is largely in line with the gradient seen in males but less pronounced. Baboons, therefore, represent a promising model to further investigate the genetic foundation of variation in aggressiveness in primates.

### Aims of this study

We investigated the *5-HTTLPR* and *MAOALPR* genotypes in baboons to test whether interspecific differences in allele frequencies correlate with variation in aggressiveness. It appears that baboon species vary quantitatively rather than qualitatively in behavior. We, therefore, predicted that different baboon species carry the same alleles but in different frequencies (Prediction 1), and that *P. papio* and *P. ursinus* are the most distinct species as concerns their genotypes (Prediction 2), as they seem to represent opposite extremes in aggressive behavior [5]. Comparable data on aggressiveness are only available for a few populations of each species. Also, it is unknown whether some geographically close populations of different species exhibit more similar levels of aggressiveness. We, therefore, included samples from different populations of each species to investigate whether allele frequencies change gradually from southern populations to northern populations, or discretely among species. Because comparable behavioral data are not available for most of these populations, we made no specific prediction as to whether this change is gradual or discrete.

Finally, we compared the *5-HTTLPR* and *MAOALPR* genotypes of baboons with those of other papionin species to obtain further insights into evolutionary changes within these well-known promoter polymorphisms over the last 8 million years among papionin species and geographic regions. To do so, we used published sequence information from other species and, in the case of *5-HTTLPR*, we analyzed additional samples from other papionin species. Because species of this tribe are relatively closely related (*Macaca* split from other papionin species only about 8 mya [71]), we expected to find similar alleles in all papionin species (Prediction 3). More specifically, for 5-HTTLPR, we expected to find variation at the PL2, whereas for *MAOALPR*, we expected to observe alleles previously found in *Macaca* and *Theropithecus*, with 5, 6, and 7 copies of the 18 bp repeat [37, 38].

## Methods

### Ethical statement

Blood samples from zoos in Germany were taken during routine health checks by experienced veterinarians. Blood samples from *P. anubis* in Tanzania were obtained from a study of disease in *P. anubis* [72]. Tissue samples from *P. papio* in Senegal were obtained from a previous study on social behavior and relatedness in *P. papio* [64]. Blood samples from *P. hamadryas* in Saudi Arabia were obtained from a previous study on the evolution of *P. hamadryas* [73]. No blood or tissue samples were collected specifically for this study. Fecal samples were collected non-invasively. All research complied with protocols approved by the Animal Welfare Body of the German Primate Center (Deutsches Primatenzentrum, DPZ) in Germany, and adhered to the legal requirements of the countries from which samples were obtained. The study was carried out in compliance with the principles of the American Society of Primatologists for the ethical treatment of non-human primates (https://www.asp.org/society/resolutions/EthicalTreatmentOfNonHumanPrimates.cfm). No animals were sacrificed or harmed for this study.

### Animals

Five species of baboons were analyzed for variation in *5-HTTLPR* and *MAOALPR*. In total, 123 wild baboons from five species were screened, including two populations of each species (Additional file 1: Table S1). We analyzed 31 samples from *P. papio* (15 females, 16 males), including 23 samples from the western (Senegal and western Guinea) and eight samples from the eastern part of the species’ distribution (eastern Guinea and Boucle du Baoulé National Park, Mali [74]); 32 samples from *P. hamadryas* (11 females, 21 males), comprising 20 African (Eritrea and Ethiopia), and 12 Arabian samples (Saudi Arabia); 21 samples from *P. anubis* (8 females, 13 males), including 10 East African (Lake Manyara National Park in Tanzania) [72] and 11 West African samples (Gashaka-Gumti National Park in Nigeria); 21 samples from *P. cynocephalus* (8 females, 13 males), all samples from Tanzania [75], including nine samples from individuals belonging to the northern mitochondrial (mt) clade and 12 samples from individuals of the southern mt clade; 18 samples from *P. ursinus* (3 females, 15 males), including 13 northern *P. ursinus* from Botswana (Moremi Game Reserve), and five southern *P. ursinus* from the Cape region and around Drakensberg, RSA.

Samples included blood, tissue, and feces. Genotypes of all samples were determined for *5-HTTLPR,* while *MAOALPR* was assessed for 98 samples (see below). Additionally, samples were analyzed for *5-HTTLPR* from one mandrill (*Mandrillus sphinx*), one drill (*Mandrillus leucophaeus*), one golden-bellied mangabey (*Cercocebus chrysogaster*), and one black mangabey (*Lophocebus aterrimus*) originating from zoos in Germany.

### Genotyping

All samples not provided as DNA were extracted from feces and tissue using the Gen-ial all-tissue DNA-kit (GEN-IAL, Troisdorf, Germany) following the standard protocol with some modifications: (1) 10 μL of 1 M DTT were added before the first incubation; (2) samples were incubated on a thermo block at 65 °C/600 rpm for 60 min followed by overnight incubation at 37 °C/300 rpm; and (3) on the day after the first centrifugation step, a maximum of 1000 μL of the supernatant was transferred into a new tube and 80 % vol. chloroform was added, briefly mixed by hand, centrifuged for 10 min at 13,000 rpm, and the upper phase was transferred into a new tube to which 75 % vol. of Lyse 3 was added.

In a first step for *5-HTTLPR* genotyping, high-quality (*i.e.* tissue or blood) samples were amplified using primers that amplified this locus in other Papionini (stpr5, 5′-GGCGTTGCCGCTCTGAATGC-3′; stpr3, 5′-GAGGGACTGAGCTGGACAACCAC-3′; amplicon size ~700 bp) [21]. After obtaining sequences for baboons, new primers were designed using AmplifX ver. 1.6.3 [76]. These primers (p*5-HTTLPR*f, 5′-CTCTGAATGCCAGCACCTAACC-3′; p*5-HTTLPR*r, 5′-AGGGGAGATAATGAGGGTGCAA-3′) amplify shorter fragments of 255/277 bp, including the entire PL2. This enabled the genotyping of low-quality DNA samples (*i.e.* feces).

For *MAOALPR* genotyping, primers described for other Papionini were used (*MAOA*-jrwF2, 5′-AGAAGGGCTGCGGGAAGC-3′; *MAOA*-jrwR, 5′-GTGCTCCACTGGGAACTGG-3′; amplicon sizes 423/441/459 bp in baboons) [37], and then primers amplifying shorter fragments of 377/395/413 bp in baboons were designed (p*MAOALPR*f, 5′-GGCTGCGGGAAGCAGAACA-3′; p*MAOALPR*r, 5′-CCACTCAGAACGGATGCTCCATT-3′). Due to the length and characteristics (*i.e.* very similar repeats) of this repeat region, designing primers that amplify shorter fragments was not possible. This explains the drop-out of several samples for this locus; the amplification of nuclear DNA fragments of ~400 bp from fecal DNA was sometimes not achievable, most likely due to degradation [77].

All PCR reactions were conducted in a 30 μL PCR-mix (1x reaction buffer, 0.16 mM for each dNTP, 0.33 μM for each primer, 1 U BiothermTaq 5000 [Genecraft, Germany], and 0.6 mg/ml BSA). We used ~50 ng of DNA per reaction for tissue and blood samples. As the determination of target-species-DNA concentration in extracts from fecal samples is very labor-intensive [78], we started with 1 μL of extract and increased incrementally to 2 μL and 4 μL (in case the initial amplification was not successful).

The thermo cycler setting for stpr-primers included (1) an initial denaturation step at 94 °C for 2 min followed by (2) 35 cycles of 94 °C – 60 °C – 72 °C, each step for 60 s. and (3) a final elongation at 72 °C for 5 min. For p*5-HTTLPR*-primer, (1) the initial denaturation step was at 94 °C for 2 min followed by (2) 35–45 cycles (depending on sample quality) of 94 °C – 62 °C – 72 °C, each step for 30 s, and (3) a final elongation at 72 °C for 5 min. For jrw-primers, (1) the initial denaturation step at 94 °C for 2 min was followed by (2) 35 cycles of 94 °C – 65 °C – 72 °C, each step 60 s, followed by (3) a final elongation at 72 °C for 5 min. For p*MAOALPR*-primers, (1) an initial denaturation step at 94 °C for 2 min was followed by (2) 40–50 cycles of 94 °C – 70 °C – 72 °C, each step 60 s, followed by (3) a final elongation at 72 °C for 5 min.

To obtain sequences, PCR products were excised from 1.0–2.5 % agarose gels, purified with the Qiagen Gel Extraction Kit (Qiagen, Germany), and sequenced on an ABI3130xL sequencer using the BigDye Terminator Cycle Sequencing Kit (Applied Biosystems, Germany). Sequences were manually checked, edited, and aligned in Bioedit ver. 7.2.3 [79]. To compare loci among species, and to identify core sequences and number of tandem repeats, we used Tandem Repeats Finder ver. 4.07b [80].

Each allele was sequenced at least once for each species. The genotypes of the remaining (non-sequenced) samples were determined by comparing the size of PCR products with fragments of known length on 2-3 % agarose gels – as commonly done in previous studies (*e.g.* [38]). Genotyping was repeated once for *5-HTTLPR* as most baboon species were monomorphic; only samples from (polymorphic) African *P. hamadryas* were genotyped three times to avoid allelic dropout [81]. Similarly, for *MAOALPR*, each allele was sequenced at least once per species. The remaining (non-sequenced) samples were genotyped by length comparisons of PCR products on agarose gels. All samples (sequenced- and non-sequenced) were genotyped three times. As males possess only one copy of the X-chromosomal *MAOA* gene, only allele frequencies (instead of genotype frequencies) are reported here for both loci (for all genotypes, see Additional file 1: Table S1). For samples derived from individuals of unknown sex, we determined the sex by a gonosomal PCR-based sexing method (C. Roos, unpublished). Sex determination was repeated once for each of these samples.

We used Cochran-Mantel-Haenszel Tests (function mantelhaen.test in R ver. 3.1.2; [82]) to detect differences in allele frequencies among species, using populations as different strata. This test indicates whether species generally differ in allele frequencies. The same test was used to conduct post-hoc comparisons between species, and Bonferroni corrected the alpha value to 0.005 as 10 species comparisons were calculated. To avoid false conclusions based upon varying number of samples from different populations, the number of alleles in the population with the larger sample size was reduced to the number of alleles in the population with smaller sample size. The original proportion of specific alleles was retained by calculating: total number of alleles in smaller population x number alleles A in larger population/total number of alleles in larger population.

## Results

### 5-HTTLPR

**Fig. 2:**
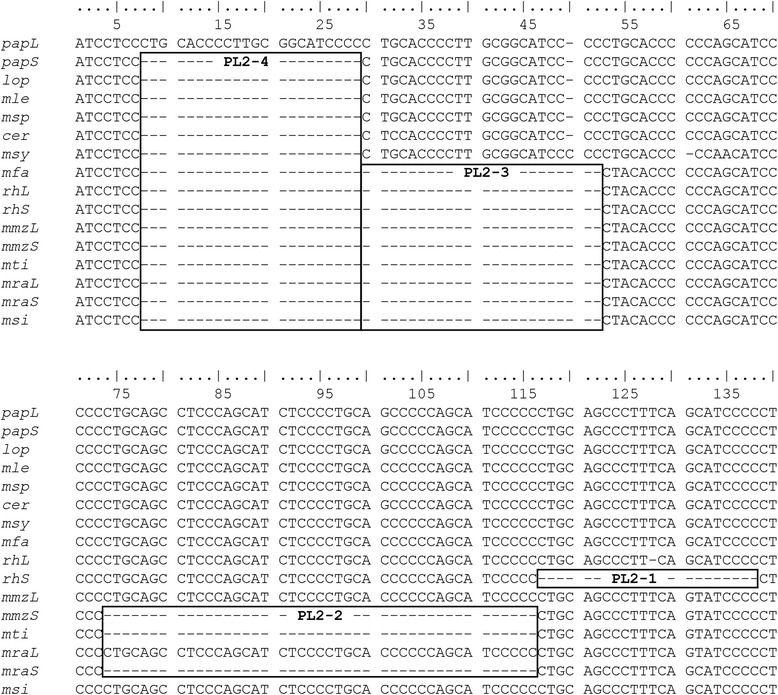
Sequence alignment of the 5-HTTLPR polymorphic locus 2 (PL2) in Papionini. The core sequence of the repeat is 5’-CTGCACCCCTCCCAGCATCTCCC-3′ but consecutive repeats can vary considerably. Within this locus, Papionini differ at four locations labelled as PL2-1 – PL2-4. This results in several alleles. This study: *papL* = long allele in *Papio hamadryas*; *papS* = short allele in baboons; *lop* = allele in *Lophocebus aterrimus*; *mle* = allele in *Mandrillus leucophaeus*; *msp* = allele in *Mandrillus sphinx*; *cer* = allele in *Cercocebus chrysogaster*. Former studies: *msy* = allele in *Macaca sylvanus*; *mfa* = allele in *M. fascicularis*; *rhL, rhS* = long and short alleles in *M. mulatta*; *mmzL* and *mmzS* = long and short alleles in *M. munzala*; *mti* = allele in *M. thibetana*; *mraL* and *mraS* = long and short alleles in *M. radiata; msi* = allele in *M. silenus*. For the complete alignment and GenBank accession numbers, see Additional file 2: Figure S1

The *5-HTTLPR* genotype in baboons consists of multiple repeats with the core sequence 5′-CTGCACCCCTCCCAGCATCTCCC-3′. There is, however, considerable variation among consecutive repeats (Additional file 2: Figure S1). There is a short allele (*papS*) with 24.2 repeats, and a long allele (*papL*) with one additional repeat in the PL2-4 (Fig. 2). The sequence of this additional repeat is identical to the adjacent repeat (at PL2-3), which is remarkable given the variation among other repeats. The *papL*-allele was only found in African *P. hamadryas*. Within this population, the allele frequency is lower than for the short allele (Table 1; *papL*: 25 %; *papS*: 75 %). All other screened baboon populations, including Arabian *P. hamadryas*, are monomorphic, carrying only the *papS*-allele. We, therefore, did not compare allele frequencies among species. Neither Prediction 1 (all baboon species carry the same alleles but in different frequencies) nor Prediction 2 (*P. papio* and *P. ursinus* are the most distinct species as concerns their genotypes) were confirmed for *5-HTTLPR*.

**Table 1:** Frequencies of *5-HTTLPR* alleles in baboons (*Papio* spp.)

					Total number of alleles	Allele frequencies			
		Number of individuals				*papL*		*papS*	
Species	Population	N	Females	Males		N	Freq.	N	Freq.
Guinea baboons *(P. papio*)	Total	31	15	16	62			62	100.0 %
	West	23	10	13	46			46	100.0 %
	East	8	5	3	16			16	100.0 %
Hamadryas baboons (*P. hamadryas*)	Total	32	11	21	64	10	15.6 %	54	84.4 %
	African	20	7	13	40	10	25.0 %	30	75.0 %
	Arabian	12	4	8	24			24	100.0 %
Olive baboons (*P. anubis*)	Total	21	8	13	42			42	100.0 %
	West African	11	2	9	22			22	100.0 %
	East African	10	6	4	20			20	100.0 %
Yellow baboons (*P. cynocephalus*)	Total	21	8	13	42			42	100.0 %
	North	9	4	5	18			18	100.0 %
	South	12	4	8	24			24	100.0 %
Chacma baboons (*P. ursinus*)	Total	18	3	15	36			36	100.0 %
	South Africa	5	2	3	10			10	100.0 %
	Moremi	13	1	12	26			26	100.0 %
Total		123	45	78	246	10	4.1 %	236	95.9 %

Despite the considerable sequence variation among different repeats of the same allele, all Papionini exhibit a high similarity at the locus (Fig. 2; Additional file 2: Figure S1), confirming Prediction 3 (there are similar alleles in all papionin species). Differences in the core sequence (5′-**CTGCA**CCCC**T**CCCAGCATC**T**CCC-3′ compared to 5′-CCCC**C**CCAGCATC**C**CCC**CTGCA**-3′ in macaques; sequence from [22] with differences highlighted) are due to the method chosen to determine the repeat sequence (see Methods). Nevertheless, there are several length differences among taxa. The *papL* allele is the longest allele described so far in Papionini and has only been detected in African *P. hamadryas*. The other four non-*Papio* papionin species assessed in this study (*Mandrillus sphinx*, *Mandrillus leucophaeus*, *Cercocebus chrysogaster, and Lophocebus aterrimus*) carry an allele similar to the *papS*-allele and the allele described for *Macaca sylvanus* (*msy*-allele) [38].

*5-HTTLPR* in all other macaque species lacks a 23 bp-repeat at PL2-3 (Fig. 2), resulting in shorter alleles. *Macaca mulatta* carries two alleles, one lacking only the repeat in PL2-3 (*rhL)*, and a shorter allele, which additionally lacks a repeat of 21/23 bp at PL2-1 (*rhS*) [21]. The *rhS*-allele has, so far, only been detected in *M. mulatta*, but several other macaque species carry an allele similar to *rhL* [38, 45]: crab-eating macaque (*M. fascicularis*; *mfa*), stump-tailed macaque (*M. arctoides*; not shown), Arunachal macaque (*M. munzala*; *mmzL*), bonnet macaque (*M. radiata*; *mraL*), Tonkean macaque (*M. tonkeana*; not shown), pig-tailed macaque (*M. nemestrina*; not shown), and lion-tailed macaque (*M. silenus*; *msi*). *Macaca radiata* and *M. munzala* carry an additional allele that lacks two repeats comprising 43 bp at PL2-2 (*mraS* and *mmzS*). Finally, the only allele found in Tibetan macaque (*M. thibetana*; *mti*) is similar to these two alleles. As sample sizes for *M. thibetana* and *M. arctoides* were small (three and two individuals, respectively) [38], it is possible that other alleles exist in these species. Taken together, papionin species differ at four sites within PL2; baboons vary only at one site (PL2-4), while macaques show considerable length variation and vary at three sites (PL2-1, PL2-2, and PL2-4).

### MAOALPR

**Fig. 3:**
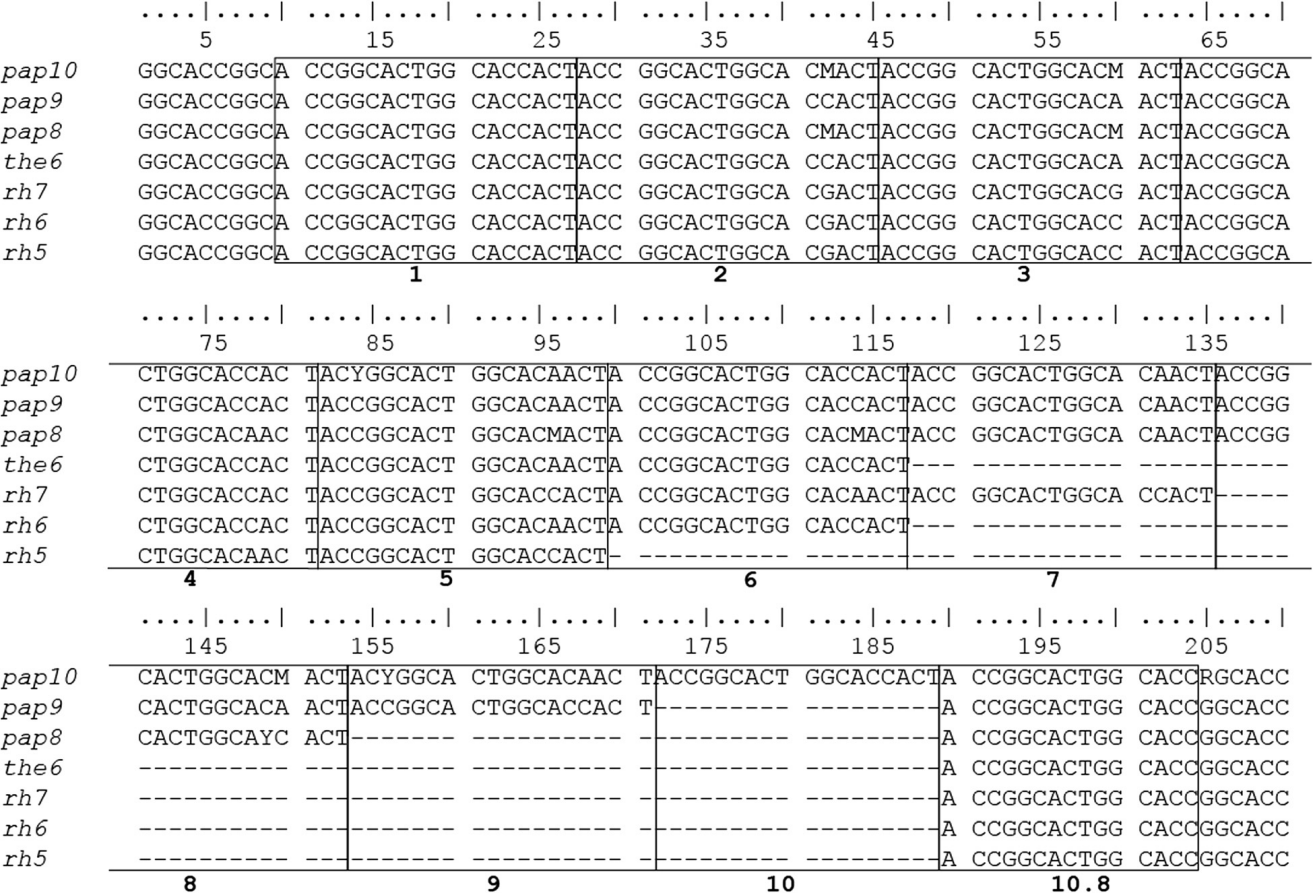
Sequence alignment of *MAOALPR* in Papionini. Number of repeats indicated below. All species of *Papio* carry alleles longer than alleles found in *M. mulatta. Theropithecus gelada*, which is more closely related to *Papio* than to *Macaca*, carries an allele similar to the rh6-allele of *M. mulatta*. This study: *pap10* = 10.8-repeat allele (*P. cynocephalus*: KJ494409; *P. anubis*: KJ494408); *pap9 =* 9.8-repeat allele (*P. ursinus*: KJ494410; *P. cynocephalus*: KJ494411, *P. anubis*: KJ494412, *P. hamadryas*: KJ494413; *P. papio*: KJ494414); *pap8 =* 8.8-repeat allele (*P. ursinus*: KJ494415; *P. cynocephalus*: KJ494416; *P. anubis*: KJ494417; *P. hamadryas*: KJ494418). Former studies: *the6* = 6.8-repeat allele in *T. gelada* (AY615803.1); *rh5*, *rh6*, *rh7* = 5.8- (JN207466.1) 6.8- (JN207467.1), and 7.8-repeat (JN207468.1) alleles in *M. mulatta*

*MAOALPR* in baboons consists of a variable number of repeats with the core sequence 5′-ACYGGCACTGGCAYVACT-3′. Alleles with 8.8 (*pap8*), 9.8 (*pap9*), and 10.8 (*pap10*) repeats were detected (Fig. 3). In contrast to *5-HTTLPR*, there is little variation in nucleotide composition among consecutive repeats (Fig. 3).

Confirming Prediction 1 (different baboon species carry the same alleles but in different frequencies) for *MAOALPR*, baboons show significant differences in allele frequencies among species (Cochran-Mantel-Haenszel test: *M*_8_^2^ = 38.9184, P < 0.001; Table 2, Fig. 1): *Papio papio* carries only the *pap9*-allele. *Papio hamadryas* carries mainly the *pap9*-allele (84.4 %) but some individuals also carry the *pap8*-allele (15.6 %). *Papio anubis* carries the *pap8*-allele and the *pap9*-allele in similar frequencies (46.2 % and 42.3 %, respectively), while the *pap10*-allele is less common (11.5 %). We observed considerable differences, however, among populations. West African *P. anubis* carries only the *pap9*-allele, while in East African *P. anubis* the *pap8*-allele is much more common (75.0 %) compared to the *pap9*-allele (6.3 %) or *pap10-*allele (18.8 %). *Papio cynocephalus* mainly carries the *pap8*-allele (52.9 %) and equal frequencies of the *pap9-*allele and the *pap10*-allele (23.5 % each). Again, populations differ considerably; in northern *P. cynocephalus* the frequency of the *pap8-*allele is high (72.7 %) while the *pap9-*allele and the *pap10*-allele frequencies are much lower (18.2 % and 9.1 %, respectively). In southern *P. cynocephalus*, the *pap10*-allele is the most common allele (50 %) and the other two alleles are less frequent (*pap8*: 16.7 %; *pap9*: 33.3 %). *Papio ursinus* shows the highest frequency of the *pap8*-allele (65 %) and low frequency of the *pap9*-allele (35 %). As in *P. anubis* and *P. cynocephalus,* populations differ considerably; northern *P. ursinus* exclusively carries the shorter *pap8*-allele, while southern *P. ursinus* exclusively carries the *pap9-*allele.

**Table 2:** Frequencies of *MAOALPR* alleles in baboons (*Papio* spp.)

					Total number of alleles^a^	Allele frequencies					
		Number of individuals				*pap8*		*pap9*		*pap10*	
Species	Population	N	Females	Males		N	Freq.	N	Freq.	N	Freq.
Guinea baboons (*P. papio*)	Total	26	13	13	39			39	100.0 %		
	West	19	9	10	28			28	100.0 %		
	East	7	4	3	11			11	100.0 %		
Hamadryas baboons (*P. hamadryas*)	Total	24	8	16	32	5	15.6 %	27	84.4 %		
	African	12	4	8	16	5	31.3 %	11	68.8 %		
	Arabian	12	4	8	16			16	100.0 %		
Olive baboons (*P. anubis*)	Total	18	8	10	26	12	46.2 %	11	42.3 %	3	11.5 %
	West African	8	2	6	10			10	100.0 %		
	East African	10	6	4	16	12	75.0 %	1	6.3 %	3	18.8 %
Yellow baboons (*P. cynocephalus*)	Total	13	4	9	17	9	52.9 %	4	23.5 %	4	23.5 %
	North	8	3	5	11	8	72.7 %	2	18.2 %	1	9.1 %
	South	5	1	4	6	1	16.7 %	2	33.3 %	3	50.0 %
Chacma baboons (*P. ursinus*)	Total	17	3	14	20	13	65.0 %	7	35.0 %		
	South Africa	5	2	3	7			7	100.0 %		
	Moremi	12	1	11	13	13	100.0 %				
Total		98	36	62	134	39	29.1 %	88	65.7 %	7	5.2 %

^a^ *MAOA* is located on the X-chromosome

Post-hoc tests (including populations as strata) indicate that *P. papio* differs significantly from *P. anubis* (*M*_2_^2^ = 17.74, *p* < 0.001), *P. cynocephalus* (*M*_2_^2^ = 20.92, *p* < 0.0001), and *P. ursinus* (χ_1_^2^ = 13.26, *p* < 0.001; note that the test indicates *M*_2_^2^ for comparisons including three alleles, but χ_1_^2^ if the compared populations only carry two alleles). *Papio hamadryas* differs significantly only from *P. cynocephalus* (M_2_^2^ = 16.42, *p* < 0.001), while no significant differences were detected among *P. anubis*, *P. cynocephalus* and *P. ursinus* (all *p* > 0.09). These results seem to confirm Prediction 2 (*P. papio* and *P. ursinus* are the most distinct species as concerns their genotypes), but given the variation between both populations of *P. ursinus*, this result is difficult to interpret (see Discussion).

**Fig. 4:**
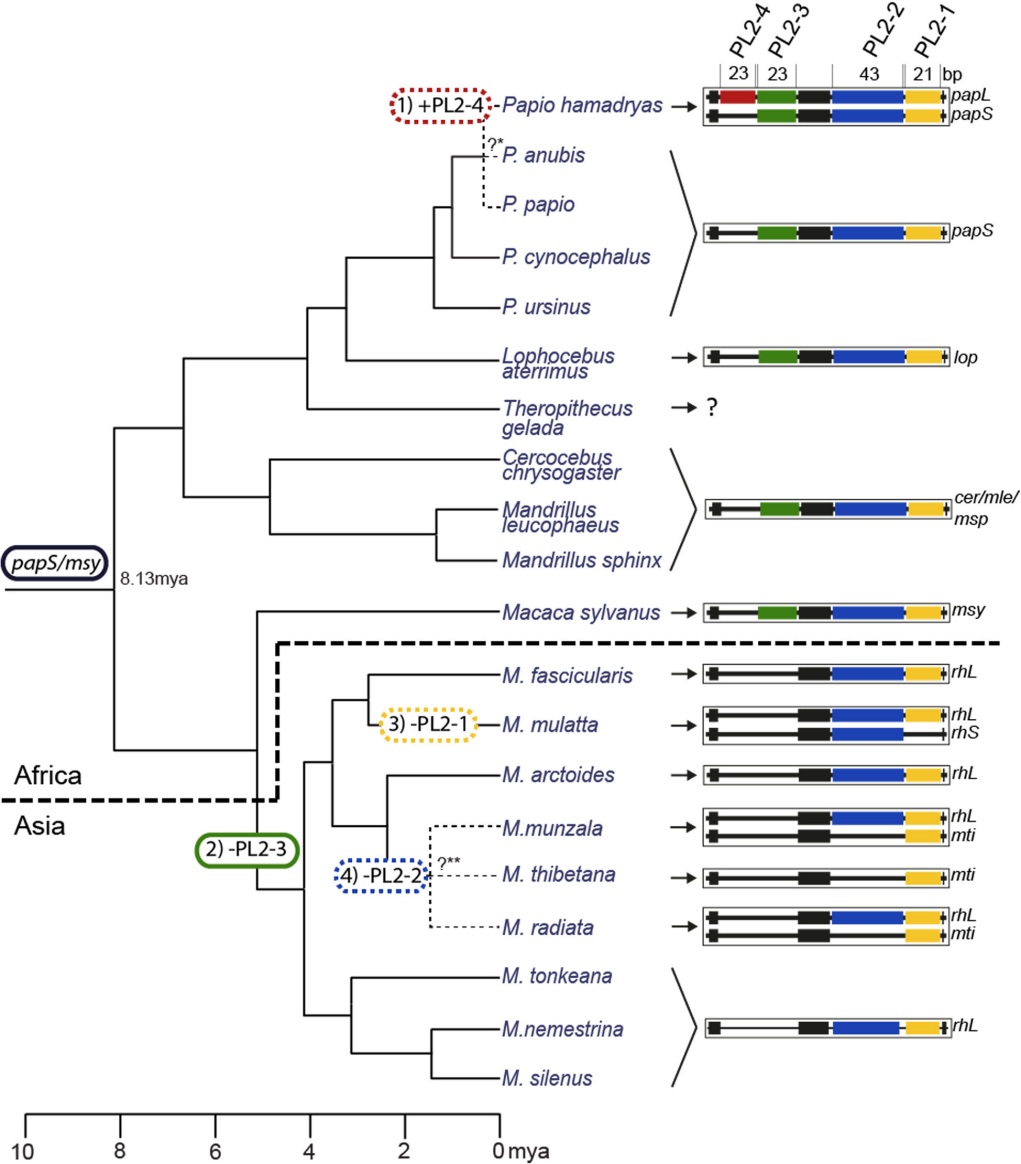
Distribution and presumed evolutionary history of alleles in *5-HTTLPR* in Papionini. Shown are hypothesized positions of insertions/deletions (indels) of repeats within the polymorphic locus 2 (PL2) in the phylogenetic tree for Papionini. Partial indels are encircled by dashed lines and complete indels by solid lines. The results of this study indicate that an allele similar to *msy* and *papS/lop/cer/mle/msp* represents the ancestral state of the locus for all Papionini, and that the following four changes occurred during the last 8 million years (for details see Discussion): (1) The additional repeat at PL2-4 (red) in the *papL-*allele represents an insertion that only emerged recently in some African *P. hamadryas*. (2) After the split of *M. sylvanus*, the ancestor of all other species of *Macaca* lost the repeat at PL2-3 (green), probably during or after their dispersal to Asia. (3) The partial loss of the repeat at PL2-1 (yellow) occurred in *M. mulatta* after the split from other macaque species. (4) The deletion of two repeats at the PL2-2 (blue) occurred in the common ancestor of the *M. sinica*-group of macaques, which includes *M. munzala*, *M. thibetana*, and *M. radiata*. Phylogenetic tree and divergence ages adapted from [71], and modified for *Papio* from [83]. *The phylogenetic relationship between *P. anubis*, *P. hamadryas* and *P. papio* is still debated [83]. **Comparable data for *M. munzala* and *M. radiata* are lacking but both species belong, together with *M. thibetana*, to the *M. sinica*-group of macaques [85]

Compared with available data from other Papionini, the *MAOALPR* of baboons shows the same core sequence (Fig. 3). Notably, alleles of baboons are longer than those of other papionin species: macaques carry only five (*rh5*), six (*rh6*), or seven (*rh7*) repeats. *Macaca mulatta* carries all three of these alleles, *M. fascicularis*, *M. nemestrina,* and *M. tonkeana* carry the *rh6-*allelle and *rh7-*allele, *M. thibetana* and *M. arctoides* only the *rh7-*allele, and *M. sylvanus* only the *rh6-*allele [37]. Geladas (*Theropithecus gelada*), which are more closely related to baboons than to macaques (Fig. 4), carry alleles with six repeats, which is more similar to macaques. Therefore, Prediction 3 (there are similar alleles in all papionin species) is partly confirmed; the repeat sequence is highly similar but the number of repeats is higher in *Papio* species than in all other papionin species.

## Discussion

In this study, we analyzed variation in *5-HTTLPR* and *MAOALPR* genotypes in five species of baboons, which exhibit pronounced interspecific variation in aggressiveness. Almost no variation in *5-HTTLPR* was detected. This indicates that the observed behavioral differences in baboons are not related to variation at this locus. Comparison with other papionin species indicates that this locus is much more variable among Asian macaques than among African Papionini. As discussed below, this may indicate differential selection pressure on *5-HTTLPR* during or after the dispersal of macaques into Asia (though genetic drift may have also played a role). In contrast, there is large variation in the distribution of *MAOALPR* alleles in baboons. In some cases, however, intraspecific variation is as large as interspecific variation. Comparable behavioral data at the population level are required to resolve the role of *MAOALPR* alleles in behavioral differences among baboon populations and species.

### 5-HTTLPR

Four of the five baboon species are monomorphic in *5-HTTLPR*. Only some African (but no Arabian) *P. hamadryas* carry a second allele. Accordingly, the genotype of this locus cannot be associated with interspecific behavioral variation. As expected, however, all investigated papionin species show variation within the same area of this polymorphism, more specifically at PL2. In comparison with previous studies, these results give some insights into evolutionary changes in *5-HTTLPR* in Papionini. Given the existence and supposed functionality of *5-HTTLPR* in many primates, such comparisons may help to better understand the evolution of promoter regions.

Wendland et al. [38] suggest that the *msy*-allele in *M. sylvanus* represents the ancestral allele in macaques. The present study confirms this assumption. Comparing alleles of 19 papionin species with respect to the evolutionary history of this tribe [71, 83, 84], suggests that the *msy*-/*papS*-allele represents the ancestral state of the locus for all Papionini. To date, this is the most parsimonious explanation as it only assumes four changes during the last 8 million years (Fig. 4):

1. As mentioned above, sequences of tandem repeats in this region are highly variable but the additional repeat at PL2-4 in the longest allele in Papionini, *papL*, is identical in sequence to the adjacent repeat (Additional file 2: Figure S1). This points towards a recent insertion of the PL2-4 repeat (Fig. 4, in red). Thus, the *papL-*allele probably emerged recently in some African *P. hamadryas*.
2. After the split of *M. sylvanus*, other macaque species lost the repeat at PL2-3 (Fig. 4, in green), resulting in the *rhL*-allele. Interestingly, the other macaque species all occur in Asia, while *M. sylvanus* and all other Papionini (with the exception of some *P. hamadryas* in Arabia) live in Africa where the tribe is thought to have its origin [50]. Thus, Asian macaques probably lost the repeat at PL2-3 during their dispersal into Asia.
3. The *rhS*-allele only occurs in *M. mulatta*. Thus, the partial loss of the repeat at PL2-1 (Fig. 4, in yellow) probably occurred after the split from other macaque species.
4. Finally, *mti*-like alleles only occur in *M. munzala*, *M. thibetana*, and *M. radiata*, all of which belong to the *Macaca sinica* group [50, 85]. Thus, the deletion of two repeats at the PL2-2 (Fig. 4, in blue) probably occurred in the common ancestor of these three species.

Overall, this could indicate that selective pressures acting on this locus differ between Asian and African papionin populations. Interestingly, the short allele in humans (which differs at PL1) seems to be the derived version of the long human allele [86], and accordingly in humans, too, the long allele seems to be the ancestral version of the polymorphism. Furthermore, frequencies of the short allele vary among human populations. This variation cannot be solely explained by demographic factors; rather, selective pressures need to be considered [86]. The geographical allele distribution in humans [87], hereby, superficially resembles the distribution in papionin species; shorter alleles have a lower frequency in African (~15-30 %) than in Asian populations (~70-80 %), while European populations show intermediate frequencies (~40-50 %). Whether the short allele confers an advantage requires further investigations [88]. In macaques, having several alleles in this genotype has been suggested to be beneficial in highly variable habitats [44, 45] (for an alternative hypothesis considering variation in social competition levels instead of variation in habitat, see [89]). To our knowledge, however, this hypothesis on the advantage of polymorphic populations in highly variable habitats has not been properly tested and comparisons of the variability of ecological conditions in Asian and African papionin habitats are not available. How range expansions by papionin species (also by humans and apes; see [90]) into new, ecologically highly variable, habitats relate to specific selective pressures and evolutionary dynamics may be a fruitful avenue for research.

Alternatively, the distribution of *5-HTTLPR* alleles could be a result of random processes (*i.e.* genetic drift). Testing for selection signals, as commonly done in coding regions, is unfortunately much more complicated in promoter regions [19]. Due to the (nearly) universal genetic code, synonymous and non-synonymous mutations in coding regions are easily inferred from the sequence, while comparative information on promoter properties (*e.g.* transcription factor binding sites) must be assessed experimentally.

### MAOALPR

*MAOALPR* allele frequencies differ significantly among some of the species, particularly between *P. papio*, which only carries the *pap9*-allele, and *P. ursinus*, *P. cynocephalus*, and *P. anubis*, which all show high frequencies of the *pap8*-allele. Inter-specific differences are, however, largely dependent on the inclusion (or exclusion) of specific populations because some of them show considerable intraspecific variation.

If populations are considered separately, do *MAOALPR* allele frequencies change gradually from south to north, and does this pattern fit with what we know about variation in aggressiveness in baboons? Generally, it appears that frequencies of the *pap8*-allele decrease and frequencies of the *pap9*-allele increase from southern populations (*P. ursinus* in Moremi) to north-western (all *P. papio*) and north-eastern populations (all *P. hamadryas*). While *P. papio* and *P. hamadryas* show some similarities in their behavior (*e.g.* male bonds, male-male greetings, and strong intersexual bonds), *P. ursinus* from Moremi differed fundamentally from *P. papio* from Senegal in male aggressiveness and tolerance [5]. In accordance with the gradual change in allele frequencies, East African *P. anubis* mainly carries the *pap8*-allele while West African *P. anubis,* which lives geographically closer to *P. papio*, only carries the *pap9*-allele. These two populations of *P. anubis* show differences in their social organization [91], but it is not known whether the western population is more similar in aggressiveness to *P. papio* than to the eastern population.

Two populations do not conform to the gradual change in allele frequencies. First, southern *P. cynocephalus* mainly carries the *pap10*-allele, which is only found in the two *P. cynocephalus* populations and in East African *P. anubis*. Second, South Africa *P. ursinus* carries only the *pap9*-allele*.* As such, it is similar in its *MAOALPR* genotype to *P. papio,* but like its conspecifics from Moremi, South Africa *P. ursinus* shows several indications of intense male contest competition [92–94]. Nevertheless, directly comparable data on aggressiveness are not yet available and, accordingly, it is not possible, at this time, to link the *MAOALPR* genotype to variation in aggressiveness among baboon populations.

Another important question is the functionality of the *MAOALPR* in baboons. Studies on humans [35], apes [90, 95], and macaques [36] indicate that variation at the *MAOALPR* genotype has an effect on transcriptional activity. Corresponding gene expression studies for baboons are still lacking but such studies are required in order to understand the role of this polymorphism in baboon behavior. Importantly, MAOA is involved in other monoamine neurotransmitter systems in addition to 5-HT, while 5-HT transporter activity only affects the 5-HT system. For example, MAOA also metabolizes noradrenalin (NA) and dopamine (DA), and both of these monoamines appear to play an important role in the expression of aggression (reviews in [13, 96–98]).

Despite these limitations, it appears that comparative data on monoamine neurotransmitter levels from different baboon populations would be informative with respect to the link between differential MAOA activity and behavior. So far, such data are only available for some *P. hamadryas* and *P. anubis* from Ethiopia [99, 100]. The authors measured cerebrospinal fluid (CSF) levels of 5-HIAA (the metabolite of 5-HT), homovanillic acid (HVA; the metabolite of DA), and 3-methoxy-4-hydroxyphenylglycol (MHPG; the metabolite of NA). While they could not detect a significant difference in 5-HIAA levels, adult male *P. hamadryas* showed higher levels of HVA and MHPG than adult male *P. anubis*. Therefore, behavioral variation in baboons could be associated with differential activity of monoamine neurotransmitters other than 5-HT, potentially regulated by differences in MAOA availability as a result of a given *MAOALPR* genotype.

## Conclusion

The results of this study suggest that there is no corresponding variation in behavior and *5-HTTLPR* in baboons. Nevertheless, comparisons of alleles among 19 papionin species indicate differences among African and Asian species that are potentially linked to geographic differences in selective pressures on this locus. *MAOALPR*, on the other hand, shows considerable interspecific and intraspecific variation in allele frequencies. Therefore, further information on behavioral variation at the population level is needed to investigate whether the genotype of this so-called ’warrior gene’ may play a role in behavioral variation in baboons. In addition to comparisons at the population level, future studies should investigate the effect of different alleles on transcription profiles, protein levels, neurotransmitter levels, and individual behavior within polymorphic populations of baboons. While this explorative study represents one of the first steps to investigate the genetics underlying variation in aggressiveness among baboon species and populations, future studies should examine sequence variation in coding and non-coding regions of those genes encoding for other proteins involved in neurotransmitter systems.

## Additional files

Additional file 1: Table S1. Sample locations and genotypes. (PDF 37 kb) Additional file 2: Figure S1. Complete alignment of *5-HTTLPR* in Papionini. Sequences from this study (with GenBank-Accession numbers): *papS(PU)* = short allele in *P. ursinus* (KJ494398); *papS(PC)* = short allele in *P. cynocephalus (*KJ494399); *papS(PA)* = short allele in *P. anubis (*KJ494400); *papS(PH)* = short allele in *P. hamadryas* (KJ494401); *papL(PH)* = long allele in *P. hamadryas* (KJ494402); *papS(PP)* = short allele in *P. papio (*KJ494403); *lop* = allele from *Lophocebus aterrimus* (KJ494404); *mle* = allele from *Mandrillus leucophaeus* (KJ494405); *msp* = allele from *Mandrillus sphinx* (KJ494406); *cer* = allele from *Cercocebus chrysogaster* (KJ494407). Sequences described for *Macaca* spp. originating from other studies: *msy* = allele *M. sylvanus* (AY897212.1); *mfa* = allele in *M. fascicularis (*EF126284.1*)*; *rhL, rhS* = long (AF191557.1) and short (Lesch et al. 1997; Wendland et al. 2006) alleles in *M. mulatta*; *mmzL* and *mmzS* = long (HM114278.1) and short (HM114279.1) alleles in *M. munzala*; *mti* = allele in *M. thibetana* (AY897213.1); *mraL* and *mraS* = long (HM114280.1) and short (HM114281.1) alleles in *M. radiata; msi* = allele in *M. silenus* (HM114282.1). (RTF 919 kb)
