# Supplementary material for: Insights into the genetic foundation of aggression in Papio and the evolution of two length-polymorphisms in the promoter regions of serotonin-related genes (5-HTTLPR and MAOALPR) in Papionini
Source: BMC Evol Biol. 2016 Jun 10;16:121. doi: 10.1186/s12862-016-0693-1 (PMC4901440; doi:10.1186/s12862-016-0693-1)
Supplement: Additional file 1: Table S1. — Sample locations and genotypes. (PDF 37 kb) [file 12862_2016_693_MOESM1_ESM.pdf]

Table S1: Sample locations and genotypes

Species	Country	Population	Location	Letter on map	*MAOALPR*	*5HTTLPR*	Sex	Internal ID
Papio papio	Guinea	West	Fouta Djallon	A	9/9	ps/ps	F	PP-248
P. papio	Guinea	West	Fouta Djallon	A	no	ps/ps	M	PP-249
P. papio	Guinea	West	Fouta Djallon	A	no	ps/ps	M	PP-254
P. papio	Guinea	West	Fouta Djallon	A	no	ps/ps	F	PP-255
P. papio	Guinea	West	Fouta Djallon	A	no	ps/ps	M	PP-269
P. papio	Guinea	West	Fouta Djallon	A	9	ps/ps	M	PP-279
P. papio	Guinea	West	Fouta Djallon	A	9	ps/ps	M	PP-281
P. papio	Guinea	West	Fouta Djallon	A	9	ps/ps	M	PP-291
P. papio	Guinea	West	Fouta Djallon	A	9/9	ps/ps	F	PP-292
P. papio	Guinea	West	Boké	A	9/9	ps/ps	F	PP-293
P. papio	Senegal	West	Niokolo Koba	A	9	ps/ps	M	PP-001
P. papio	Senegal	West	Niokolo Koba	A	9	ps/ps	M	PP-002
P. papio	Senegal	West	Niokolo Koba	A	9	ps/ps	M	PP-005
P. papio	Senegal	West	Niokolo Koba	A	9	ps/ps	M	PP-007
P. papio	Senegal	West	Niokolo Koba	A	9/9	ps/ps	F	PP-009
P. papio	Senegal	West	Niokolo Koba	A	9/9	ps/ps	F	PP-011
P. papio	Senegal	West	Niokolo Koba	A	9	ps/ps	M	PP-015
P. papio	Senegal	West	Niokolo Koba	A	9	ps/ps	M	PP-027
P. papio	Senegal	West	Niokolo Koba	A	9/9	ps/ps	F	PP-029
P. papio	Senegal	West	Niokolo Koba	A	9/9	ps/ps	F	PP-034
P. papio	Senegal	West	Niokolo Koba	A	9/9	ps/ps	F	PP-045
P. papio	Senegal	West	Niokolo Koba	A	9/9	ps/ps	F	PP-056
P. papio	Senegal	West	Niokolo Koba	A	9	ps/ps	M	PP-246
P. papio	Guinea	East	Kankan	B	9/9	ps/ps	F	PP-283
P. papio	Mali	East	Boucle du Baoué	B	9/9	ps/ps	F	PP-187
P. papio	Mali	East	Boucle du Baoué	B	9/9	ps/ps	F	PP-188
P. papio	Mali	East	Boucle du Baoué	B	9/9	ps/ps	F	PP-189
P. papio	Mali	East	Boucle du Baoué	B	9	ps/ps	M	PP-190
P. papio	Mali	East	Boucle du Baoué	B	no	ps/ps	F	PP-205
P. papio	Mali	East	Boucle du Baoué	B	9	ps/ps	M	PP-206
P. papio	Mali	East	Boucle du Baoué	B	9	ps/ps	M	PP-207
P. hamadryas	Eritrea	African	Af Himbol	G	no	ps/ps	M	PH-E205
P. hamadryas	Eritrea	African	Af Himbol	G	9/9	ps/ps	F	PH-E206
P. hamadryas	Eritrea	African	Af Himbol	G	no	ps/ps	F	PH-E207
P. hamadryas	Eritrea	African	Af Himbol	G	no	ps/ps	M	PH-E208
P. hamadryas	Eritrea	African	Af Himbol	G	9	ps/ps	M	PH-E209
P. hamadryas	Eritrea	African	Filfil Bridge	G	9/9	ps/ps	F	PH-E236
P. hamadryas	Eritrea	African	Filfil Bridge	G	no	ps/ps	M	PH-E237
P. hamadryas	Eritrea	African	Filfil Bridge	G	no	ps/pl	F	PH-E238
P. hamadryas	Eritrea	African	Durfo	G	no	ps/ps	F	PH-E240
P. hamadryas	Eritrea	African	Durfo	G	no	ps/pl	M	PH-E241
P. hamadryas	Eritrea	African	Durfo	G	9/9	ps/pl	F	PH-E243
P. hamadryas	Ethiopia	African	Gerba Luku	G	9	ps/pl	M	PH-E310
P. hamadryas	Ethiopia	African	Gerba Luku	G	8	ps/pl	M	PH-E311
P. hamadryas	Ethiopia	African	Gerba Luku	G	8	pl/pl	M	PH-E312
P. hamadryas	Ethiopia	African	Gerba Luku	G	9	ps/ps	M	PH-E313

Species	Country	Population	Location	Letter on map	*MAOALPR*	*5HTTLPR*	Sex	Internal ID
P. hamadryas	Ethiopia	African	Gerba Luku	G	no	ps/pl	M	PH-E314
P. hamadryas	Ethiopia	African	Gerba Luku	G	8	ps/ps	M	PH-E315
P. hamadryas	Ethiopia	African	Gerba Luku	G	8	ps/pl	M	PH-E316
P. hamadryas	Ethiopia	African	Gerba Luku	G	9/9	ps/ps	F	PH-E318
P. hamadryas	Ethiopia	African	Gerba Luku	G	8	ps/pl	M	PH-E319
P. hamadryas	Saudi Arabia	Arabian	Abha	H	9/9	ps/ps	F	PH-A01
P. hamadryas	Saudi Arabia	Arabian	Abha	H	9/9	ps/ps	F	PH-A02
P. hamadryas	Saudi Arabia	Arabian	Baha	H	9	ps/ps	M	PH-A03
P. hamadryas	Saudi Arabia	Arabian	Al Akhal	H	9	ps/ps	M	PH-A04
P. hamadryas	Saudi Arabia	Arabian	Taif	H	9	ps/ps	M	PH-A05
P. hamadryas	Saudi Arabia	Arabian	Taif	H	9	ps/ps	M	PH-A06
P. hamadryas	Saudi Arabia	Arabian	Abha	H	9	ps/ps	M	PH-A07
P. hamadryas	Saudi Arabia	Arabian	Abha	H	9/9	ps/ps	F	PH-A08
P. hamadryas	Saudi Arabia	Arabian	Baha	H	9/9	ps/ps	F	PH-A09
P. hamadryas	Saudi Arabia	Arabian	Baha	H	9	ps/ps	M	PH-A10
P. hamadryas	Saudi Arabia	Arabian	Al Akhal	H	9	ps/ps	M	PH-A11
P. hamadryas	Saudi Arabia	Arabian	Al Akhal	H	9	ps/ps	M	PH-A12
P. anubis	Nigeria	West African	Gashaka	C	9	ps/ps	M	PA-EY16
P. anubis	Nigeria	West African	Gashaka	C	9	ps/ps	M	PA-EY17
P. anubis	Nigeria	West African	Gashaka	C	9/9	ps/ps	F	PA-EY18
P. anubis	Nigeria	West African	Gashaka	C	9	ps/ps	M	PA-EY19
P. anubis	Nigeria	West African	Gashaka	C	no	ps/ps	M	PA-EY21
P. anubis	Nigeria	West African	Gashaka	C	9	ps/ps	M	PA-EY23
P. anubis	Nigeria	West African	Gashaka	C	no	ps/ps	M	PA-EY24
P. anubis	Nigeria	West African	Gashaka	C	9	ps/ps	M	PA-EY25
P. anubis	Nigeria	West African	Gashaka	C	no	ps/ps	M	PA-EY26
P. anubis	Nigeria	West African	Gashaka	C	9	ps/ps	M	PA-EY27
P. anubis	Nigeria	West African	Gashaka	C	9/9	ps/ps	F	PA-EY28
P. anubis	Tanzania	East African	Lake Manyara	D	9	ps/ps	M	PA-S03
P. anubis	Tanzania	East African	Lake Manyara	D	10/10	ps/ps	F	PA-S04
P. anubis	Tanzania	East African	Lake Manyara	D	8	ps/ps	M	PA-S05
P. anubis	Tanzania	East African	Lake Manyara	D	8/10	ps/ps	F	PA-S06
P. anubis	Tanzania	East African	Lake Manyara	D	8/8	ps/ps	F	PA-S07
P. anubis	Tanzania	East African	Lake Manyara	D	8	ps/ps	M	PA-S08
P. anubis	Tanzania	East African	Lake Manyara	D	8	ps/ps	M	PA-S09
P. anubis	Tanzania	East African	Lake Manyara	D	8/8	ps/ps	F	PA-S10
P. anubis	Tanzania	East African	Lake Manyara	D	8/8	ps/ps	F	PA-S11
P. anubis	Tanzania	East African	Lake Manyara	D	8/8	ps/ps	F	PA-S12
P. cynocephalus	Tanzania	North	Mikumi	E	8	ps/ps	M	PC-0951
P. cynocephalus	Tanzania	North	Mikumi	E	9	ps/ps	M	PC-0954
P. cynocephalus	Tanzania	North	North of Morogoro	E	8	ps/ps	M	PC-0961
P. cynocephalus	Tanzania	North	North of Morogoro	E	8/8	ps/ps	F	PC-0962
P. cynocephalus	Tanzania	North	North of Morogoro	E	no	ps/ps	F	PC-0963
P. cynocephalus	Tanzania	North	Dodoma-->Iringa	E	8	ps/ps	M	PC-0978
P. cynocephalus	Tanzania	North	Dodoma-->Iringa	E	8/8	ps/ps	F	PC-0981
P. cynocephalus	Tanzania	North	Dodoma-->Iringa	E	9/10	ps/ps	F	PC-0983
P. cynocephalus	Tanzania	North	Dodoma-->Iringa	E	8	ps/ps	M	PC-0988

Species	Country	Population	Location	Letter on map	*MAOALPR*	*5HTTLPR*	Sex	Internal ID
P. cynocephalus	Tanzania	South	Kilewa->Kibiti	F	9	ps/ps	M	PC-0985
P. cynocephalus	Tanzania	South	Iringa-->Makambako	F	8	ps/ps	M	PC-0991
P. cynocephalus	Tanzania	South	100km North of Songea	F	10	ps/ps	M	PC-1002
P. cynocephalus	Tanzania	South	North of Namtumbo	F	no	ps/ps	M	PC-1017
P. cynocephalus	Tanzania	South	Kilimansela	F	no	ps/ps	M	PC-1024
P. cynocephalus	Tanzania	South	Chem-Chem	F	no	ps/ps	F	PC-1027
P. cynocephalus	Tanzania	South	Chem-Chem	F	no	ps/ps	M	PC-1028
P. cynocephalus	Tanzania	South	Tunduru->Masasi	F	no	ps/ps	F	PC-1042
P. cynocephalus	Tanzania	South	Tunduru->Masasi	F	no	ps/ps	M	PC-1045
P. cynocephalus	Tanzania	South	West of Masasi	F	10/10	ps/ps	F	PC-1057
P. cynocephalus	Tanzania	South	Chiwata	F	no	ps/ps	F	PC-1063
P. cynocephalus	Tanzania	South	Nanguruwe	F	9	ps/ps	M	PC-1073
P. ursinus	Botswana	Moremi	Moremi Game Reserve	I	8	ps/ps	M	PU-0024
P. ursinus	Botswana	Moremi	Moremi Game Reserve	I	8	ps/ps	M	PU-0025
P. ursinus	Botswana	Moremi	Moremi Game Reserve	I	8	ps/ps	M	PU-0026
P. ursinus	Botswana	Moremi	Moremi Game Reserve	I	8	ps/ps	M	PU-0028
P. ursinus	Botswana	Moremi	Moremi Game Reserve	I	8	ps/ps	M	PU-0029
P. ursinus	Botswana	Moremi	Moremi Game Reserve	I	8	ps/ps	M	PU-0050
P. ursinus	Botswana	Moremi	Moremi Game Reserve	I	8	ps/ps	M	PU-0067
P. ursinus	Botswana	Moremi	Moremi Game Reserve	I	8	ps/ps	M	PU-0069
P. ursinus	Botswana	Moremi	Moremi Game Reserve	I	no	ps/ps	M	PU-0074
P. ursinus	Botswana	Moremi	Moremi Game Reserve	I	8	ps/ps	M	PU-0075
P. ursinus	Botswana	Moremi	Moremi Game Reserve	I	8/8	ps/ps	F	PU-0079
P. ursinus	Botswana	Moremi	Moremi Game Reserve	I	8	ps/ps	M	PU-0114
P. ursinus	Botswana	Moremi	Moremi Game Reserve	I	8	ps/ps	M	PU-0120
P. ursinus	South Africa	South African	Olifantskop/Cape Region	J	9	ps/ps	M	PU-1441
P. ursinus	South Africa	South African	Cape Town/Cape Region	J	9/9	ps/ps	F	PU-U04
P. ursinus	South Africa	South African	Cape Town/Cape Region	J	9/9	ps/ps	F	PU-U11
P. ursinus	South Africa	South African	Newcastle/Drakensberg	J	9	ps/ps	M	PU-1386
P. ursinus	South Africa	South African	Mt. Currie/Drakensberg	J	9	ps/ps	M	PU-1419
