# Supplementary material for: Insights into the genetic foundation of aggression in Papio and the evolution of two length-polymorphisms in the promoter regions of serotonin-related genes (5-HTTLPR and MAOALPR) in Papionini
Source: BMC Evol Biol. 2016 Jun 10;16:121. doi: 10.1186/s12862-016-0693-1 (PMC4901440; doi:10.1186/s12862-016-0693-1)
Supplement: Additional file 2: Figure S1. — Complete alignment of 5-HTTLPR in Papionini. Sequences from this study (with GenBank-Accession numbers): papS(PU) = short allele in P. ursinus (KJ494398); papS(PC) = short allele in P. cynocephalus (KJ494399); papS(PA) = short allele in P. anubis (KJ494400); papS(PH) = short allele in P. hamadryas (KJ494401); papL(PH) = long allele in P. hamadryas (KJ494402); papS(PP) = short allele in P. papio (KJ494403); lop = allele from Lophocebus aterrimus (KJ494404); mle = allele from Mandrillus leucophaeus (KJ494405); msp = allele from Mandrillus sphinx (KJ494406); cer = allele from Cercocebus chrysogaster (KJ494407). Sequences described for Macaca spp. originating from other studies: msy = allele M. sylvanus (AY897212.1); mfa = allele in M. fascicularis (EF126284.1); rhL, rhS = long (AF191557.1) and short (Lesch et al. 1997; Wendland et al. 2006) alleles in M. mulatta; mmzL and mmzS = long (HM114278.1) and short (HM114279.1) alleles in M. munzala; mti = allele in M. thibetana (AY897213.1); mraL and mraS = long (HM114280.1) and short (HM114281.1) alleles in M. radiata; msi = allele in M. silenus (HM114282.1). (RTF 919 kb) [file 12862_2016_693_MOESM2_ESM.rtf]

Figure S1

                    10        20        30        40        50        60        70        80        90       100                  
           ....|....|....|....|....|....|....|....|....|....|....|....|....|....|....|....|....|....|....|....|
papS(PU)   CCTGCTGCAGCCCTCCCAGCATcTCCCTGTACCCCTCCTAGGATCTCCCCTGCA-CCCCCCATTATCCTCC----------------------CTGCACC 
papS(PC)   CCTGCTGCAGCCCTCCCaGCATCTCCCTGTACCCCTCCTAGGATCTCCCCTGCA-CCCCCCATTATCCTCC----------------------CTGCACC 
papS(PA)   CCTGCTGCAGCCCTcCCAGCATCTCCCTGTACCCCTCCTAGGATCTCCCCTGCA-CCCCCCAtTATCCTCC----------------------CTGCACC 
papS(PH)   CCTGCTGCAGCCCTCTCAGCATCTCCCTGTACCCCTCCTAGGATCTCCCCTGCA-CCCCCCATTATCCTCC----------------------CTGCACC 
papL(PH)   CCTGCTGCAGCCCTCCCAGCATCTCCCTGTACCCCTCCTAGGATCTCCCCTGCA-CCCCCCATTATCCTCCCTGCACCCCTTGCGGCATCCCCCTGCACC 
papS(PP)   CCTGCTGCAGCCCTCCCAGCATCTCCCTGTACCCCTCCTAGGATCTCCCCTGCA-CCCCCCATTATCCTCC----------------------CTGCACC 
lop        CCTGCTGCAGCCCTCCCAGCATCTCCCTGTACCCCTCCTAGGATCTCCCCTGCA-CCCCCCATTATCCTCC----------------------CTGCACC 
mle        CCTGCTGCAGCCCTCCCAGCATCTCCCTGTACCCCTCCTAGGATCTCCCCTGCA-CCCCCCATTATCCTCC----------------------CTGCACC 
msp        CCTGCTGCAGCCCTCCCAGCATCTCCCTGTACCCCTCCTAGGATCTCCCCTGCA-CCCCYCATTATCCTCC----------------------CTGCACC 
cer        CCTGCTGCAGCCCTCCCAGCATCTCCCTGTACCCCTCCTAGGATCTCCCCTGCACCCCCCCATTATCCTCC----------------------CTCCACC 
msy        CCTGCTGCAGCCCTCCCAGCATCTCCCTGTACCCCTCCTAGGATCTCCCCTGCA-CCCCCCATTATCCTCC----------------------CTGCACC 
mfa        CCTGCTGCAGCCCTCCCAGCATCTCCCTGTACCCCTCCTAGGATCTCCCCTGCA-CCCCCCATTATCCTCC----------------------------- 
rhL        CCTGCTGCAGCCCTCCCAGCATCTCCCTGTACCCCTCCTAGGATCTCCCCTGCAACCCCC-ATTATCCTCC----------------------------- 
rhS        CCTGCTGCAGCCCTCCCAGCATCTCCCTGCACCCCTCCTAGGATCTCCCCTGCA-CCCCCCATTATCCTCC----------------------------- 
mmzL       CCTGCTGCAGCCCTCCCAGCATCTCCCTGTACCCCTCCTAGGATCTCCCCTGCA-CCCCC-ATTATCCTCC----------------------------- 
mmzS       CCTGCTGCAGCCCTCCCAGCATCTCCCTGTACCCCTCCTAGGATCTCCCCTGCA-CCCCC-ATTATCCTCC----------------------------- 
mti        CCTGCTGCAGCCCTCCCAGCATCTCCCTGTACCCCTCCTAGGATCTCCCCTGCA-CCCCC-ATTATCCTCC----------------------------- 
mraL       CCTGCTGCAGCCCTCCCAGCATCTCCCTGTACCCCTCCTAGGATCTCCCCTGCA-CCCCC-ATTATCCTCC----------------------------- 
mraS       CCTGCTGCAGCCCTCCCAGCATCTCCCTGTACCCCTCCTAGGATCTCCCCTGCA-CCCCCCATTATCCTCC----------------------------- 
msi        CCTGCTGCAGCCCTCCCAGCATCTCCCTGTACCCCTCCTAGGATCTCCCCTGCA-CCCCCCATTATCCTCC----------------------------- 

                   110       120       130       140       150       160       170       180       190       200         
           ....|....|....|....|....|....|....|....|....|....|....|....|....|....|....|....|....|....|....|....|
papS(PU)   CCTTGCGGCATCC-CCCTGCACCCCCCAGCATCCCCCCTGCAGCCTCCCAgCATCTCCCCTGCAGCCCCCAGCATCCCCCCTGCAGCCCTTTCAGCATCC 
papS(PC)   CCTTGCGGCATCC-CCCTGCACCCCCCAGCATCCCCCCTGCAGCCTCCCAGCATCTCCCCTGCAgCCCCCAGCATCCCCCCTGCAgCCCtTTCAGCATCC 
papS(PA)   CCTTGCGGCATCC-CCCTGCACCCCCCAGCATCCCCCCTGCAGCCTCCCAGCATCTCCCCTGCAGCCCCCAGCATCCCCCCTGCAgCCCTTTCAGCATCC 
papS(PH)   CCTTGCGGCATCC-CCCTGCACCCCCCAGCATCCCCCCTGCAGCCTCCCAGCATCTCCCCTGCAGCCCCCAGCATCCCCCCTGCAGCCCTTTCAGCATCC 
papL(PH)   CCTTGCGGCATCC-CCCTGCACCCCCCAGCATCCCCCCTGCAGCCTCCCAGCATCTCCCCTGCAGCCCCCAGCATCCCCCCTGCAGCCCTTTCAGCATCC 
papS(PP)   CCTTGCGGCATCC-CCCTGCACCCCCCAGCATCCCCCCTGCAGCCTCCCAGCATCTCCCCTGCAGCCCCCAGCATCCCCCCTGCAGCCCTTTCAGCATCC 
lop        CCTTGCGGCATCC-CCCTGCACCCCCCAGCATCCCCCCTGCAGCCTCCCAGCATCTCCCCTGCAGCCCCCAGCATCCCCCCTGCAGCCCTTTCAGCATCC 
mle        CCTTGCGGCATCC-CCCTGCACCCCCCAGCATCCCCCCTGCAGCCTCCCAGCATCTCCCCTGCACCCCCCAGCATCCCCCCTGCAGCCCTTTCAGCATCC 
msp        CCTTGCGGCATCC-CCCTGCACCCCCCAGCATCCCCCCTGCAGCCTCCCAGCATCTCCCCTGCACCCCCCAGCATCCCCCCTGCAGCCCTTTCAGCATCC 
cer        CCTTGCGGCATCC-CCCTGCACCCCCCAGCATCCCCCCTGCAGCCTCCCAGCATCTCCCCTGCAGCCCCCAGCATCCCCCCTGCAGCCCTTTCAGCATCC 
msy        CCTTGCGGCATCCCCCCTGCACCC-CCAACATCCCCCCTGCAGCCTCCCAGCATCTCCCCTGCACCCCCCAGCATCCCCCCTGCAGCCCTTTCAGCATCC 
mfa        ----------------CTACACCCCCCAGCATCCCCCCTGCAGCCTCCCAGCATCTCCCCTGCACCCCCCAGCATCCCCCCTGCAGCCCTTTCAGCATCC 
rhL        ----------------CTACACCCCCCAGCATCCCCCCTGCAGCCTCCCAGCATCTCCCCTGCACCCCCCAGCATCCCCCCTGCAGCCCTT-CAGCATCC 
rhS        ----------------CTACACCCCCCAGCATCCCCCCTGCAGCCTCCCAGCATCTCCCCTGCACCCCCCAGCATCCCCC-------------------- 
mmzL       ----------------CTACACCCCCCAGCATCCCCCCTGCAGCCTCCCAGCATCTCCCCTGCACCCCCCAGCATCCCCCCTGCAGCCCTTTCAGTATCC 
mmzS       ----------------CTACACCCCCCAGCATCCCCC-------------------------------------------CTGCAGCCCTTTCAGTATCC 
mti        ----------------CTACACCCCCCAGCATCCCCC-------------------------------------------CTGCAGCCCTTTCAGTATCC 
mraL       ----------------CTACACCCCCCAGCATCCCCCCTGCAGCCTCCCAGCATCTCCCCTGCACCCCCCAGCATCCCCCCTGCAGCCCTTTCAGTATCC 
mraS       ----------------CTACACCCCCCAGCATCCCCC-------------------------------------------CTGCAGCCCTTTCAGCATCC 
msi        ----------------CTACACCCCCCAGCATCCCCCCTGCAGCCTCCCAGCATCTCCCCTGCACCCCCCAGCATCCCCCCTGCAGCCCTTTCAGCATCC 

                   210       220       230       240       250       260       270       280       290       300         
           ....|....|....|....|....|....|....|....|....|....|....|....|....|....|....|....|....|....|....|....|
papS(PU)   CCCTGCACCCCTCCCAGGATCTCCCTTGCACcCTCATTATCTCCCCTGCACCCCTCGCAGTATCCCCCCACACCTCCATTATCCCCCCTGCACCCCTTGC 
papS(PC)   CCCTGCACCCCTCCCAGgATCTCCCtTGCACCCTCATTATCTCCCCTGCACCCCTCGCAGTATCCCCCCACACCTCCATTATCCCCCCTGCACCCCTTGC 
papS(PA)   CCCTGCACCCCTCCCAGGATCTCCCTTGCACCCTCATTATCTCCCCTGCACCCCTCGCAGTATCCCCCCACACCTCCATTATCCCCCCTGCACCCCTTGC 
papS(PH)   CCCTGCACCCCTCCCAGGATCTCCCTTGCACCCTCATTATCTCCCCTGCACCCCTCGCAGTATCCCCCCACACCTCCATTATCCCCCCTGCACCCCTTGC 
papL(PH)   CCCTGCACCCCTCCCAGGATCTCCCTTGCACCCTCATTATCTCCCCTGCACCCCTCGCAGTATCCCCCCACACCTCCATTATCCCCCCTGCACCCCTTGC 
papS(PP)   CCCTGCACCCCTCCCAGGATCTCCCTTGCACCCTCATTATCTCCCCTGCACCCCTCGCAGTATCCCCCCACACCTCCATTATCCCCCCTGCACCCCTTGC 
lop        CCCTGCACCCCTCCCAGGATCTCCCTTGCACCCTCATTATCTCCCCTGCACCCCTCGCAGTATCCCCCCACACCTCCATTATCCCCCCTGCACCCCTTGC 
mle        CCCTGCACCCC-CCCAGGATCTCCCTTGCACCCCCATTATCTCCCCTGCACCCCTCGCAGTATCCCCCTGCACCTCCATTATCCCCCCTGCACCCCTTGC 
msp        CCCTGCACCCCYCCCAGGATCTCCCTTGCACCCCCATTATCTCCCCTGCACCCCTCGCAGTATCCCCCTGCACCTCCATTATCCCCCCTGCACCCCTTGC 
cer        CCCTGCACCCCTCCCAGGATCTCCCTTGCATCCCCATTATCTCCCCTGCACCCCTCGCAGTATCCCCCCGCACCTCCATTATCCCCCCTGCACCCCTTGC 
msy        CCCTGCACCCCTCCCAGGATCTCCCTTGCA-CCCCATTATCTCCCCTGCACCCCTCGCAGTATTCCCCCGCACCTCCATTATCCCCCCTGCACCCCTTGC 
mfa        CCCTGCACCCCTCCCAGGATCTCCCTTGCATCCCCATTATCTCCCCTGCACCCCTCGCAGTATCCCCCCGCACCTCCATTATCCCCCCTGCACCCCTTGC 
rhL        CCCTGCACCCCTCCCAGGATCTCCCTTGCATCCCCATTATCTCCCCTGCACCCCTCGCAGTATCCCCCCGCACCTCCATTATCCCCCCTGCACCCCTCGC 
rhS        --CTGCACCCCTCCCAGGATCTCCCTTGCATCCCCATTATCTCCCCTGCACCCCTCGCAGTATCCCCCCGCACCTCCATTATCCCCCCTGCACCCCTTGC 
mmzL       CCCTGCACCCCTCCCAGGATCTCCCTTGCATCCCCATTATCTCCCCTGCACCCCTCGCAGTATCCCCCCGCACCTCCATTATCCCCCCTGCACCCCTTGC 
mmzS       CCCTGCACCCCTCCCAGGATCTCCCTTGCATCCCCATTATCTCCCCTGCACCCCTCGCAGTATCCCCCCGCACCTCCATTATCCCCCCTGCACCCCTTGC 
mti        CCCTGCACCCCTCCCAGGATCTCCCTTGCATCCCCATTATCTCCCCTGCACCCCTCGCAGTATCCCCCCGCACCTCCATTATCCCCCCTGCACCC-TTGC 
mraL       CCCTGCACCCCTCCCAGGATCTCCCTTGCATCCCCATTATCTCCCCTGCACCCCTCGCAGTATCCCCCCGCACCTCCATTATCCCCCCTGCACCCCTTGC 
mraS       CCCTGCACCCCTCCCAGGATCTCCCTTGCATGCCCATTATCTCCCCTGCACCCCTCGCAGTATCCCCCCGCACCTCCATTATCCACCCTGCACCCCTTGC 
msi        CCCTGCACCCCTCCCAGGATCTCCCTTGCATCCCCATTATCTCCCCTGCACCCCTCTCAGTATCCCCCCGCACCTCCATTATCCCCCCTGCACCCCTTGC 

                   310       320       330       340       350       360       370       380       390       400         
           ....|....|....|....|....|....|....|....|....|....|....|....|....|....|....|....|....|....|....|....|
papS(PU)   GGCATCCCCC--TGCACCCCCCAGTATTCCCCCCTGCAGCCCCCCC-AGCATCTCCCCCGCACCG-CTCAGCATCCCCCCT-GCAGCCCTTCC-AGCATC 
papS(PC)   GGCATCCCCC--TGCACCCCCCAgtATTCCCCCCTGCACCCCCCCc~AgcATCtCCCCYGCACCG-CTCAGCATCCCCCCT~GCAgCCCTTCC-AGCATC 
papS(PA)   GGCATCCCCC--TGCACCCCCCAGTATTCCCCCCTGCAgCCCCCCC-AGCATCTCCCCCGCACCG-CTCAGCATCCCCCCT-GCAGCCCTTCC-AGCATC 
papS(PH)   GGCATCCCCC--TGCACCCCCCAGTATTCCCCCCTGCAgCCCCCCC-AGCATCTCCCCCGCACCG-CTCAGCATCCCCCCT-GCAGCCCTTCC-AGCATC 
papL(PH)   GGCATCCCCC--TGCACCCCCCAGTATTCCCCCCTGCASCCCCCCC-AGCATCTCCCCCGCACCG-CTCAGCATCCCCCCT-GCAGCCCTTCC-AGCATC 
papS(PP)   GGCATCCCCC--TGCACCCCCCAGTATTCCCCCCTGCAgCCCCCCC-AGCATCTCCCCCGCACCG-CTCAGCATCCCCCCT-GCAGCCCTTCC-AGCATC 
lop        GGCATCCCCC--TGCACCCCCCAGTATTCCCCCCTGCAGCCCCCCC-AGCATCTCCCCCGCACCG-CTCAGCATCCCCGCT-GCAGCCCTTCC-AGCATC 
mle        GGCATCCCCCC-TGCACCCCCCAGTACTCCCCC-TACAGCCCCCCC-AGCATCTCCCCCACACCG-CTCAGCATCCCCCCTAGCAGCCCTTCC-AGCATC 
msp        GGCATCCCCCC-TGCACCCCCCAGTACTCCCCC-TACAGCCCCCCC-AGCATCTCCCCCACACCG-CTCAGCATCCCCCCTAGCAGCCCTTCC-AGCATC 
cer        GGCATCCCCCC-TGCACCCCCCAGTATTCCCCC-TGCAGCCCCCCCCAGCATCTCCCCGGCACCG-CTCAGCATCCCCCCT-GCAGCCCTTCC-AGCATC 
msy        GGCATCCCCCC-TGCACCCCCCAGTATTCCCTC-TGCAGCCCCCC--AGCATCTCCCCCGCACCG-CCCAGCATCCCCCCT-GCAGCCCTTCCCAGCAAC 
mfa        GGCATCCCCCC-TGCACCCCCCAGTATTCCCCC-TGCAGCACCCCCCAGCATCTCCCCCGCACCG-CCCAGCATCCCCCCT-GCAGCCCTTCC-AGCAAC 
rhL        GGCATCCCCCC-TGCACCCTCCAGTATTCCCCC-TGCAGCACCCCCCAGCATCTCCCCCGCACCG-CCCAGCATCCCCCCT-GCAGCCCTTCC-AGCAAC 
rhS        GGCATCCCCCC-TACACCC  
mmzL       GGCATCCCCCC-TGCACCC                                                                                  
mmzS       GGCATCCCCCC-TGCACCCCCCAGTATTCCCCC-TG                                                                 
mti        GGCATCCCCCCCTGCACCC-CCAGTATTTCCCC-TGGAGCCCCCCCCAGCATCTCCCC-GCACGGACACAGCATCCCCCCT-GCAGCCCTTCCAAGCAAC 
mraL       GGCATCCCCCC-TGCACCCCCCAGTATTCCCCC-TGCAGCCCCCCCCAGCATCTCCCCCGCACCG-CTCAGCATCCCCCCT-GCAGCCCTTCCA-GCAAC 
mraS       GGCATCCCCCC-TGCACCC                                                                                  
msi        GGAATCCCCCC-TGCACCCCCCAGTATTCCCCC-TGCAGCCCCCCCCAGCATCTCCCCCGCACCG-CTCAGCATCCCCCCT-GCAGCCCTTCC-AGCAAC 

                   410       420       430       440       450       460       470       480       490       500         
           ....|....|....|....|....|....|....|....|....|....|....|....|....|....|....|....|....|....|....|....|
papS(PU)   CCCCT-GCATCCCTCCCAGGATcTCCCCTGCAACCCCATTATCAcCCCTGCACCCCTCGCGGCATCCCCCTACACCCCCCAACATACCCTCTGCACCCCC 
papS(PC)   CCCCT-GCATCCCTCCCAGGATCTCCcCTGCAACCcCATTATCACCCCTGCACCCCTCGCGGCATCCCCCTACACCCCCcAACATaCCcTCTgCaCCCCC 
papS(PA)   CCCCT-GCATCCCTCCCAGGATCTCCCCTGCAACCCCATTATCACCCCTGCACCCCTCGCGGCATCCCCCTACACCCCCCAACATACCCTCTGCACCCCC 
papS(PH)   CCCCT-GCATCCCTCCCAGGATCTCCCCTGCAACCCCATTATCACCCCTGCACCCCTCGCGGCATCCCCCTACACCCCCCAACATACCCTCTGCACCCCC 
papL(PH)   CCCCT-GCATCCCTCCCAGGATCTCCCCTGCAACCCCATTATCACCCCTGCACCCCTCGCGGCATCCCCCTACACCCCCCAACATACCCTCTGCACCCCC 
papS(PP)   CCCCt-GCATCCCTCCCAGGATCTCCCCTGCAACCCCATTATCACCCCTGCACCCCTCGCGGCATCCCCCTACACCCCCCAACATACCCTCTGCaCCCCC 
lop        CCCCT-GCATCCCTCCCAGGATCTCCCCTGCAACCCCATTATCACCCCTGCACCCCTCGCGGCATCCCCCTACACCCCCCAACATACCCTCTGCACCCCC 
mle        CCCCT-GCATCCCTCCCAGGATCTCCCCTGCAACCCCATTATCCCCCCTGCATCCCTCGCGGCATCCCCCTACACCCCCCAACATACCCTCTGCACCCCC 
msp        CCCCT-GCATCCCTCCCAGGATCTCCCCTGCAACCCCATTATCCCCCCTGCATCCCTCGCGGCATCCCCCTACACCCCCCAACATACCCTCTGCACCCCC 
cer        CCCCT-GCATCCCTCCCAGGATCTCCCCTGCAACCCCATTATCCCCCCTGCACCCCTCGCGGCATCCCCCTACACCCCCCAACATACTCTCTGCACCCCC 
msy        CCCCT-GCAT                                                                                           
mfa        CCCCT-GCATCCCTCCCAGGATCTCCCCTGCAACCCCATTATCCCCCCTGCACCCCTCGCGGCATCCCCCTACACCCCCCAACATACCCTCTGCACCCCC 
rhL        CCCCTTGCATCCCTCCCAGGATCTCCCCTGCAACCCCATTATCCCCC-TGCACCCCTCGCGGCATCCCCCTACACCCCCCAACATACCCTCTGCACCCCC 
rhS                                                                                                             
mmzL                                                                                                            
mmzS                                                                                                            
mti        CCCCT-GCAT                                                                                           
mraL       CCCCT-GCATCCCTCCCAGGATCTCCCCTGCAACCCCATTATCCCCCCTGCACCCCTCGCGGCATCCCCCTACACCCCCCAACATACCCTCTGCACCCCC 
mraS                                                                                                            
msi        CCCCT-GCATCCCTCCCAGGATCTCCCCTGCAACCCCATTATCCCCCCTGCACCCCTCGCGGCATCCCCCTACACCCCCCAACATACCCTCTGCACCCCC 

                   510       520       530       540       550       560       570       580       590       600         
           ....|....|....|....|....|....|....|....|....|....|....|....|....|....|....|....|....|....|....|....|
papS(PU)   CGGAATCCCCCCTGCACCCCTCCAGCATTCCCCTTGCACCCTCCCAGTATCCCCCTGCATCCCGGGCTCCAAGCCTCCCGCCCACCTTGCGGTCCCCGCC 
papS(PC)   CGGAATCCcCCCTGCACCCcTCCAgcATTCCCCTTGCaCCCTCCCAgtAtCCCCCTGCATCCCGGgCTCCAagCCTCCCGCCCACCTTgCggtCCCCgCC 
papS(PA)   CGGAATCCCCCCTGCACCCCTCCAGCATTCCCCTTGCACCCTCCCAGTATCCCCCTGCATCCCGGGCTCCAAGCCTCCCGCCCACCTTGCGGTCCCCGCC 
papS(PH)   CGGAATCCCCCCTGCACCCCTCCAGCATTCCCCTTGCACCCTCCCAGTATCCCCCTGCATCCCGGGCTCCAAGCCTCCCGCCCACCTTGCGGTCCCCGCC 
papL(PH)   CGGAATCCCCCCTGCACCCCTCCAGCATTCCCCTTGCACCCTCCCAGTATCCCCCTGCATCCCGGGCTCCAAGCCTCCCGCCCACCTTGCGGTCCCCGCC 
papS(PP)   CGGAATCCCCCCTGCACCCCTCCAGCATTCCCCTTGCACCCTCCCAGTATCCCCCTGCATCCCGGGCTCCAAGCCTCCCGCCCACCTTGCGGTCCCCGCC 
lop        CGGAATCCCCCCTGCACCCCTCCAGCATTCCCCTTGCACCCTCCCAGTATCCCCCTGCATCCCGGGCTCCAAGCCTCCCGCCCACCTTGCGGTCCCCGCC 
mle        CGGAATCCCCCCTGCACCCCTCCAGCATTCCCCTTGCACCCTCCCAGTATCCCCCTGCATCCCGGGCTCCAAGCCTCCCGCCCACTTTGCGGTCCCCGCC 
msp        CGGAATCCCCCCTGCACCCCTCCAGCATTCCCCTTGCACCCTCCCAGTATCCCCCTGCATCCCGGGCTCCAAGCCTCCCGCCCACTTTGCGGTCCCCGCC 
cer        CGGAATCCCCCCTGCACCCCTCCAGCATTCCCCTTGCACCCTCCCAGTATCCCCCTGCATCCCGGGCTCCAAGCCTCCCGCCCACCTTGCGGTCCCCGTC 
msy                                                                                                             
mfa        CGGAATCCCCCCTGCACCCCTCCAGCATTCCCCTTGCACCCTCCCAGTATCCCCCTGCATCCCGGGCTCCAAGCCTCCCGCCCACCTTGCGGTCCCCGCC 
rhL        -GGAATCCCCCCTGCACCCCTCCAGCATTCCCCTTGCACCCTCCCAGTATCCCCCTGCATCCCGGGCTCCAAGCCTCCCGCCCACCTTGCGGTCCCCGCC 
rhS                                                                                                             
mmzL                                                                                                            
mmzS                                                                                                            
mti                                                                                                             
mraL       CGGAATCCCCCCTGCACCCCTCCAGCATTCCCCTTGCACCCTCCCAGTATCCCCCTGCATCCCGGGCTCCAAGCCTCCCGCCCACCTTGCGGTCCCCGCC 
mraS                                                                                                            
msi        CGGAATCCCCCCTGCACCCCTCCAGCATTCCCCTTGCACCCTCCCAGTATCCCCCTGCATCCCGGGCTCCAAGCCTCCCGCCCACCTTGCGGTCCCCGCC 

                   610       620       630       640       650       660       670       680       690        
           ....|....|....|....|....|....|....|....|....|....|....|....|....|....|....|....|....|....|.
papS(PU)   CTGGCGTCTAGGTGGCACCAGAATCCCGCGCGGACTCCTCCCGCTGGGTGCCGCCCT-CGCTTGCCCGTGGTTGTCCAGCTCAGTCCCTC  
papS(PC)   CTGGCgTcTAgGTGGCACCaGAATCCCGCGCGGACtCCTCCCgCTggGtgccgccct-cgcttgcccgtggttgtccagctcagtccctc  
papS(PA)   CTGGCGTCTAGGTGGCACCAGAATCCCGCGCGGACTCCTCCCGCTGGGTGCCGCCCT-CGCTTGCCCGTGGTTGTCCAGCTCAGTCCCTC  
papS(PH)   CTGGCGTCTAGGTGGCACCAGAATCCCGCGCGGACTCCTCCCGCTGGGTGCCGCCCT-CGCTTGCCCGTGGTTGTCCAGCTCAGTCCCTC- 
papL(PH)   CTGGCGTCTAGGTGGCACCAGAATCCCGCGCGGACTCCTCCCGCTGGGTGCCGCCCT-CGCTTGCCCGTGGTTGTCCAGCTCAGTCCCTC  
papS(PP)   CTGGCGTCTAGGTGGCACCAGAATCCCGCGCGGACTCCTCCCGCTGGGTGCCGCCCT-CGCTTGCCCGTGGTTGTCCAGCTCAGTCCCTC  
lop        CTGGCGTCTAGGTGGCACCAGAATCCCGCGCGGACTCCTCCCGCTGGGTGCCGCCCT-CGCTTGCCCGTGGTTGTCCAGCTCAGTCCCTC  
mle        CTGGCGTCTAGGTGGCACCAGAATCCCGCGCGGACTCCTCCCGCTGGGTGCCGCCCT-CGCTTGCCCGTGGTTGTCCAGCTCAGTCCCTC  
msp        CTGGCGTCTAGGTGGCACCAGAATCCCGCGCGGACTCCTCCCGCTGGGTGCCGCCCT-CGCTTGCCCGTGGTTGTCCAGCTCAGTCCCTC  
cer        CTGGCGTCTAGGTGGCACCAGAATCCTGCGCGGACTCCTCCCGCTGGGTGCCGCCCT-CGCTTGCCCGTGGTTGTCCAGCTCAGTCCCTC  
msy                                                                                                    
mfa        CTGGCGTCTAGGTGGCACCAGAATCCCGCGCGGACTCCTCCCGCTGGGTGCCGCCCT-CGCTTGCCCGTGGTTGTCCAGCTCAGTCCCTC  
rhL        CTGGCGTCTAGGTGGCACCAGAATCCCGCGCGGACTCCTCCCGCTGGGTGCCGCCCT-CGCTTGCCCG                        
rhS                                                                                                    
mmzL                                                                                                   
mmzS                                                                                                   
mti                                                                                                    
mraL       CTGGCGTCTAGGTGGCACCAGAATCCCGCGCGGACTCCTCCCGCTGGGTGCCGCCCT-CGCTTGCCC                         
mraS                                                                                                   
msi        CTGGCGTCTAGGTGGCACCAGAATCCCGCGCGGACTCCTCCCGCTGGGTGCCGCCCTTCGCTTGCCC                         


Figure S1: Complete alignment of 5-HTTLPR in Papionini. Sequences from this study (with GenBank-Accession numbers): papS(PU) = short allele in P. ursinus (KJ494398); papS(PC) = short allele in P. cynocephalus (KJ494399); papS(PA) = short allele in P. anubis (KJ494400); papS(PH) = short allele in P. hamadryas (KJ494401); papL(PH) = long allele in P. hamadryas (KJ494402); papS(PP) = short allele in P. papio (KJ494403); lop = allele from Lophocebus aterrimus (KJ494404); mle = allele from Mandrillus leucophaeus (KJ494405); msp = allele from Mandrillus sphinx (KJ494406); cer = allele from Cercocebus chrysogaster (KJ494407).
Sequences described for Macaca spp. originating from other studies: msy = allele M. sylvanus (AY897212.1); mfa = allele in M. fascicularis (EF126284.1); rhL, rhS = long (AF191557.1) and short (Lesch et al. 1997; Wendland et al. 2006) alleles in M. mulatta; mmzL and mmzS= long (HM114278.1) and short (HM114279.1) alleles in M. munzala; mti = allele in M. thibetana (AY897213.1); mraL and mraS = long (HM114280.1) and short (HM114281.1) alleles in M. radiata; msi = allele in M. silenus (HM114282.1).
